# Global prevalence of anxiety and depression among medical students during the COVID-19 pandemic: a systematic review and meta-analysis

**DOI:** 10.1186/s40359-024-01838-y

**Published:** 2024-06-10

**Authors:** Yen-Ko Lin, Ita Daryanti Saragih, Chia-Ju Lin, Hsin-Liang Liu, Chao-Wen Chen, Yung-Sung Yeh

**Affiliations:** 1 Department of Emergency Medicine, Kaohsiung Medical University Hospital, Kaohsiung Medical University, Kaohsiung, Taiwan; 2 Division of Trauma and Surgical Critical Care, Department of Surgery, Kaohsiung Medical University Hospital, Kaohsiung Medical University, Kaohsiung, Taiwan; 3 Department of Medical Humanities and Education, College of Medicine, Kaohsiung Medical University, Kaohsiung, Taiwan; 4 Department of Emergency Medicine, College of Medicine, Kaohsiung Medical University, Kaohsiung, Taiwan; 5 Center for Medical Education and Humanizing Health Professional Education, Kaohsiung Medical University, Kaohsiung, Taiwan; 6 Doctoral student, College of Nursing, Kaohsiung Medical University, Kaohsiung, Taiwan; 7 College of Nursing, Kaohsiung Medical University, Kaohsiung, Taiwan

**Keywords:** Prevalence, Anxiety, Depression, COVID-19, Medical students

## Abstract

**Purpose:**

As future physicians, medical students have experienced tremendous pressure during the ongoing COVID-19 pandemic, which is associated with a high risk of depression and anxiety. We aimed to investigate an overview of the prevalence of anxiety and depression among medical students in various countries during the global COVID-19 pandemic, and discuss associated stressors.

**Methods:**

We systematically searched CINAHL, EMBASE, MEDLINE, PubMed, and Web of Science for relevant articles from December 1, 2019 to March 15, 2023. We performed meta-analysis using a random-effects model with REML method to calculate the pooled prevalence of anxiety and depression. Begg’s and Egger’s tests were used to identify the potential risk of publication bias. Meta-regression was used to explore potential sources of heterogeneity.

**Results:**

We identified 130 studies reporting anxiety and depression among 132,068 medical students during the COVID-19 pandemic. Eight screening tools were identified for anxiety and six for depression. The pooled prevalence of mental health outcomes for anxiety and depression was 45% (95% confidence interval [CI], 40%–49%) and 48% (95% CI, 43%–52%), respectively. The pooled prevalence of mental health outcomes for moderate and severe anxiety and moderate and severe depression was 28% (95% CI, 24%–32%) and 30% (95% CI, 26%–35%), respectively. There was high heterogeneity between studies, with I^2^ ranging from 99.58%–99.66%. Continent and survey date were included in the meta-regression model. The results of meta-regression revealed that medical students in Asia had a lower prevalence of anxiety, and depression than those in other regions. The survey date (from February to June, 2020) showed a significantly positive correlation with the prevalence of anxiety and depression.

**Conclusions:**

We demonstrated the global prevalence of anxiety and depression among medical students during the COVID-19 pandemic. The data highlight that medical students worldwide are at high risk of experiencing anxiety and depression. The reported stressors can be categorized into personal, academic, environmental and cultural, and pandemic factors. Schools and institutions should ensure optimal alternative learning environments for uninterrupted provision of medical education. The appropriate authorities should prioritize the provision of adequate protection for medical students and establish policies to promote new methods of training and education during a disaster, such as via distance learning.

**Supplementary Information:**

The online version contains supplementary material available at 10.1186/s40359-024-01838-y.

## Introduction

On March 11, 2020, the World Health Organization declared the coronavirus disease 2019 (COVID-19) global pandemic [1]. As of March 17, 2024, there have been more than 774,954,393 COVID-19 cases globally and 7,040,264 deaths [2]. To slow the rising numbers of COVID-19 infections and deaths, extraordinary scientific efforts have been made to develop vaccines against COVID-19 infection and distribute them in many countries. However, by the end of 2022, the pandemic has yet to be resolved [3]. Multiple epidemic waves of COVID-19 have been reported in many countries [4, 5], and countries worldwide must be prepared for the possibility of future waves [6].

Medical students, pre-clinical medical students, and students in clinical rotations represent a population that is vulnerable to infectious disease exposure, especially during the COVID-19 pandemic. In many countries, the medical education of students in their pre-clinical years has been changed to online activities to maintain social distancing and avoid new outbreaks of COVID-19 infection, and medical students have been advised to stay at home [7, 8]. However, in many countries, medical students in the clinical years are required to be involved in caring for patients owing to the shortage of professional health workers in hospitals because of increasing numbers of patients with COVID-19 infection [9], even though students may not have appropriate and complete training or sufficient clinical experience to be able to protect themselves and handle complex clinical situations. Therefore, both the Medical Schools Council (MSC) in the United Kingdom and the American Association of Medical Colleges (AAMC) in the United States have published a guideline for the participation of medical students as health care providers during the COVID-19 pandemic in which this group is recommended to work as volunteers [10, 11].

The effects of the COVID-19 pandemic on mental health outcomes most likely differ among different populations [3]. The emotional response to the pandemic might be stronger for groups who are vulnerable to infection, such as health care providers [12]. The ongoing pandemic has been a tremendously challenging situation for health care providers who are exposed to patients with COVID-19. Their work not only places them at high risk of becoming infected but also can lead to a fear of contagion and spread of the virus to loved ones [13]. This difficult situation has led to the development of mental health problems such as distress, anxiety, depression, insomnia, post-traumatic stress disorder (PTSD), denial, and fear among health workers [14, 15]. Furthermore, exposure to COVID-19 infection among health care providers is associated with a high risk of experiencing depression and anxiety [12]. Health care providers who are in charge of caring for patients with COVID-19 have demonstrated increased levels of distress, anxiety, and depression [16]. As future physicians, medical students may have also experienced tremendous pressure during the ongoing COVID-19 pandemic, which is associated with a high risk of depression and anxiety.

Mental health issues surrounding mental illness are prevalent among medical students [17, 18], and these might be exacerbated to become a serious issue during the global COVID-19 pandemic. Mental health problems jeopardize the well-being of medical students, leading to learning problems, which can have an impact on delivering quality patient care in the future. This is a serious problems to which medical schools and institutions should devote greater attention by developing strategies to help medical students [17]. In a cross-sectional study conducted in Bangladesh during the COVID-19 pandemic among 425 medical students, 55% experienced anxiety and 44% experienced depression [19]. Another cross-sectional study conducted among medical students in Libya revealed that 65% developed anxiety and 78% developed depression [20]. A study in the United States found that 66% of medical students had developed anxiety during the COVID-19 pandemic [16]. Two meta-analysis studies conducted during the COVID-19 pandemic revealed that 28% of medical students had developed anxiety [21] and 31% had depression [22] respectively; additionally, further meta-analysis conducted in 2023 found that 38% of medical students had developed anxiety and 41% had depression during the COVID-19 pandemic [23]. Given the high prevalence of anxiety and depression among medical students in developing and developed countries, continued efforts must be made to collect and analyze data regarding the effects of the COVID-19 pandemic on mental health outcomes among medical students to obtain a complete picture of this phenomenon globally and address this knowledge gap.

An evaluation of the psychological status will help clarify approaches for targeted psychological intervention during the ongoing COVID-19 pandemic, prompting the further development of medical and health public services. Therefore, updated evidence is crucial regarding the global mental health situation in the population of medical students during the COVID-19 pandemic. In this study, we aimed to investigate updated estimates of the prevalence of anxiety and depression among medical students during the COVID-19 pandemic, and discuss associated stressors.

## Materials and methods

This systematic review (study protocol registered on PROSPERO-CRD42021252968) was conducted following the Preferred Reporting Items for Systematic Reviews and Meta-Analyses (PRISMA) [24].

### Search strategy

Five electronic databases were used to retrieve relevant studies. The reviewing author searched the CINAHL, EMBASE, MEDLINE, PubMed, and Web of Science databases from December 1, 2019 to March 15, 2023 to identify studies published between 2020 and 2023. To avoid missing pertinent research, grey literature search using Google Scholar and manually searching by examining included studies from prior systematic reviews or meta-analysis studies were conducted. The search language was limited to English. The MeSH terms used to develop the search included: medical students; OR medical undergraduate; OR medical postgraduate; OR education, medical, undergraduate; AND mental disorder; OR mental health; OR affective disorder; OR mood disorder; OR depressive disorder; OR depression; OR anxiety; OR stress, psychological; OR depress*; OR anxiety*; OR mental wellbeing; AND COVID-19; OR coronavirus disease 2019; OR pandemic; OR 2019-nCoV; OR SARS-CoV-2; OR COV-19. Details of the search strategy are presented in Additional File 1: Appendix 1. Additionally, the references of the identified articles were searched manually and appropriate articles were reviewed.

### Eligibility criteria

The following inclusion criteria were applied: a) studies conducted among students of medicine; b) cross-sectional study, cohort study, or case–control study; c) provided outcomes of anxiety and depression; d) studies conducted related to COVID-19 pandemic; and e) published in English language. The following exclusion criteria were applied: a) review studies; and b) studies that did not provide the full text. Medical students included students who were enrolled in the M.D. program (Doctor of Medicine) and M.B.B.S. programs (Bachelor of Medicine and Bachelor of Surgery).

### Study selection and data extraction

Two authors (LYK and SID) independently screened all titles and abstracts according to the defined eligibility criteria, after removing duplicates using EndNote software. The authors used the PRISMA flow diagram to report study eligibility, and they independently followed the selection process for all studies and retrieved the full texts of those studies that passed the first-level screening. A full-text review was performed for each study, and data extraction was conducted in duplicate by two reviewers. The fields extracted were author, publication year, country where the study was conducted, continent of the study country, sample size, sex of participants, age, number and percentage of anxiety and depression among medical students, instruments used to evaluate anxiety and depression, and study period. All discrepancies were resolved in consultation with a third reviewer (LCJ).

### Study risk of bias assessment

Accurately judging and choosing the appropriate tool for each included study is an important step in analyzing the methodological quality (risk of bias) of each identified study and exploring whether the study is of low quality or has a high risk of bias [25]. For each reviewed source, two authors (LYK and SID) assessed the risk of bias using the Joanna Briggs Institute (JBI) critical appraisal checklist for studies reporting prevalence data to assess the level of the evidence; the overall quality of each study [26]. Nine dimensions include sample frame, participant recruitment, sample size, subjects and setting description, representativeness, valid methods for the identification, a standard and reliable measurement way, valid assessment of mental problems, appropriate statistical analysis, and response rate. All discrepancies were resolved in consultation with a third reviewer (LCJ).

### Statistical analysis

The pooled prevalence of anxiety and depression among medical students during the COVID-19 pandemic was calculated using a random-effects model with REML mothod. Anxiety and depression in this study referred to “anxiety symptoms” and “depressive symptoms” because most included studies used self-rated questionnaires or instruments; an actual illness could not be diagnosed using the screening tools alone. The selected studies reported the dichotomous variable of anxiety and depression as being present or absent in medical students, according to the study authors’ defined cutoff score for the selected screening instruments. Anxiety and depression were further divided into categories according to the scale of the instruments: anxiety, moderate and severe (anxiety MS) and depression, moderate and severe (depression MS). The heterogeneity of each variable in the pooled estimate was indicated with *I2* using a random-effects model. For *I2*, 25%–49% indicated low heterogeneity, 50%–74% moderate heterogeneity, and > 75% indicated high heterogeneity [27, 28]. Funnel plots and forest plots were generated for all analyses. Sensitivity analysis was performed to confirm stability and reliability. We used leave-one-out meta-analysis to identify the influence of each study on the effect-size estimates. Both Begg’s test and Egger’s tests were used to identify the potential risk of publication bias. If various levels of heterogeneity were identified among studies and in subgroup analysis, meta-regression was used to explore the heterogeneity. Moderator variables for subgroup analyses and meta-regression were chosen post hoc. A value of *p* < 0.05 was considered statistically significant. All statistical analyses were conducted using Stata version 17.0 (StataCorp LLC, College Station, TX, USA).

## Results

### Study selection

**Fig. 1:**
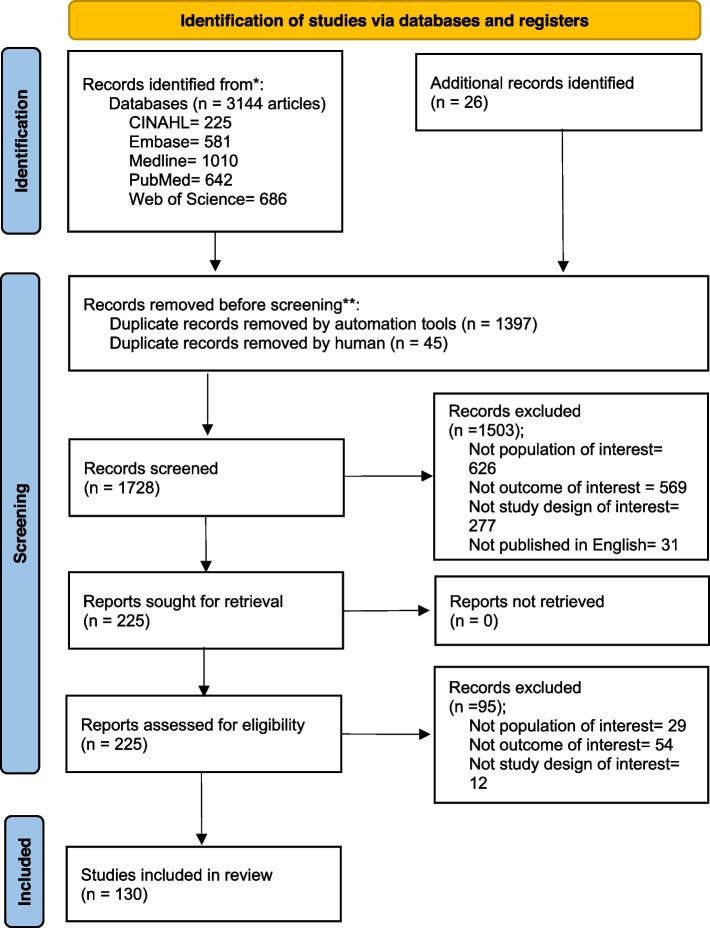
Flow chart for selection of reviewed articles. *Consider, if feasible to do so, reporting the number of records identified from each database or register searched (rather than the total number across all databases/registers). **If automation tools were used, indicate how many records were excluded by a human and how many were excluded by automation tools. From: Page MJ, McKenzie JE, Bossuyt PM, Boutron I, Hoffmann TC, Mulrow CD, et al. The PRISMA 2020 statement: an updated guideline for reporting systematic reviews. BMJ 2021;372:n71. doi: 10.1136/bmj.n71

Using the search strategy, we identified 3,144 articles in five electronic databases. After the removal of duplicates, the titles and abstracts of 1,728 articles were screened; 225 articles were determined to satisfy the eligibility criteria. After a full-text review, ninety-five articles were deemed ineligible. The remaining 130 articles were included in the final analysis. Details of the study selection process are presented in a PRISMA flow diagram (Fig. 1). The PRISMA checklist is presented in Additional File 2: Appendix 2.

### Study characteristics

All included articles were cross-sectional studies. Three of the 130 studies included multiple sets of data; therefore, a total of 135 sets of data were included in the final analysis. A total of 132,068 medical students were included. Twenty-eight studies were conducted in China [29–56]. Sixteen studies were conducted in India [57–72]. Eleven studies were conducted in Pakistan [73–83]. Ten studies were conducted in Saudi Arabia [84–93]. Nine studies were conducted in United States [16, 94–101]. Five studies were conducted in France [102–106] and Turkey [107–111]. Four studies each were conducted in Brazil [112–115], Malaysia [116–119], and Peru [120–123]. Three studies were conducted in Indonesia [124–126] and Thailand [127–129]. Two studies each were conducted in Bangladesh [19, 130], Egypt [131, 132], Korea [133, 134], Morocco [135, 136], Nepal [137, 138], Spain [139, 140], and multi-countries [141, 142]. One study each was conducted in Germany [143], Greece [144], Iran [145], Iraq [146], Italy [147], Japan [148], Jordan [149], Kazakhstan [150], Mexico [151], Slovak [152], Sudan [153], Tunisia [154], United Arab Emirates [155], and Vietnam [156]. Furthermore, according to continent or region, seventy-six studies were conducted in Asia, nineteen studies in the Middle East, eleven studies were conducted in Europe, ten studies in North America, eight studies in South America, and Five study was conducted in Africa. The average age of included participants ranged from 17 to 26.1 years. The prevalence of anxiety in 100 studies ranged from 3.8% to 96.1%, and the prevalence of depression in 97 studies ranged from 7.5% to 99.6%. The prevalence of anxiety MS in 82 studies ranged from 2.3% to 73.7%, and the prevalence of depression MS in 78 studies ranged from 1.2% to 90.2%. The study periods of the included publications were between 2019 and May 2022. A summary of study characteristics is presented in Table 1.

**Table 1:** Summary of selected studies on mental health outcomes among medical students during COVID-19 outbreak

No	Author/year	Country	Continent	Sample size	Female	Age	Medical student				Instrument use		
						(mean)	Anxiety	Anxiety MS^a^	Depression	Depression MS^b^	Anxiety	Depression	Survey period
							N (%)	N (%)	N (%)	N (%)	(m, MS)^c^	(m, MS)^d^	
1	AbuDujain et al., 2021 [84]	Saudi Arabia	Middle East	345	174	24.5	115 (33.3)	48 (13.9)	152 (44.1)	71 (20.6)	GAD-7	PHQ-9	3/2020 ~ 10/2020
											(5, 10)	(5, 10)	
2	Adhikari et al., 2021 [137]	Nepal	Asia	223	88	NA	NA	NA	139 (62.3)	52 (23.3)	NA	PHQ-9	8/2020 ~ 9/2020
												(5, 10)	
3	Aftab et al., 2021 [141]	Pakistan, India, and Saudi Arabia	Asia and Middle East	418	272	NA	NA	158 (37.8)	386 (92.3)	173 (41.5)	GAD-7	PHQ-9	NA
											(5, 10)	(5, 10)	
4	Ahmed et al., 2022 [73]	Pakistan	Asia	312	208	NA	78 (25)	NA	164 (52.6)	NA	DASS-21	DASS-21	9/2021
											(-, -)	(-, -)	
5	Al-Hasani et al., 2021 [146]	Iraq	Middle East	870	604	NA	707 (81.3)	641 (73.7)	647 (74.4)	539 (62.0)	DASS-21	DASS-21	11/20/2020 ~ 1/2/2021
											(8, 10)	(10, 14)	
6	Ali et al., 2020 [74]	Pakistan	Asia	182	116	NA	152 (83.5)	128 (70.3)	152 (83.5)	126 (69.2)	GAD-7	PHQ-9	5/2020
											(5, 10)	(5, 10)	
7	Alkhamees et al., 2020 [85]	Saudi Arabia	Middle East	305	161	NA	NA	NA	NA	153 (50.2)	NA	PHQ-9	2/2020 ~ 3/2020
												(5, 10)	
8	Alkwai, 2021 [86]	Saudi Arabia	Middle East	53	26	23.9	NA	18 (34)	NA	14 (26)	GAD-7	PHQ-9	4/22/2020
											(-, 8)	(-, 10)	
9	Allah et al., 2021 [87]	Saudi Arabia	Middle East	1591	715	23.4	945 (59.4)	306 (19.2)	NA	NA	GAD-7	NA	5/22/2020 ~ 6/22/2020
											(5, 10)		
10	Almarri et al., 2022 [88]	Saudi Arabia	Middle East	7116	3583	NA	5114 (71.8)	2880 (56.3)	NA	NA	GAD-7	NA	9/2020 ~ 11/2020
											(5, 10)		
11	Almutairi A., Jahan S., 2022 [89]	Saudi Arabia	Middle East	179	179	22.5	NA	48 (26.8)	NA	NA	BAI	NA	8/2021 ~ 12/2021
											(-, 22)		
12	Alrashed et al., 2022 [90]	Saudi Arabia	Middle East	361	215	NA	149(41.3)	NA	42(11.6)	NA	DASS-21	DASS-21	12/2021 ~ 1/2022
											(8, 10)	(10, 14)	
13	Alrashed et al., 2021 [91]	Saudi Arabia	Middle East	463	207	NA	NA	NA	249 (53.8)	95 (20.5)	NA	PHQ-9	NA
												(5, 10)	
14	Aolymat et al., 2023 [149]	Jordan	Middle East	385	385	19.9	205 (53.2)	164 (42.6)	206 (53.5)	57 (14.8)	DASS-21	DASS-21	1/2021 ~ 2/2021
											(8, 10)	(10, 14)	
15	Avila-Carrasco et al., 2022 [151]	Mexico	North America	728	427	NA	495 (67.9)	NA	592 (81.3)	NA	GADS	GADS	3/2021
											(4, -)	(2, -)	
16	Batais et al., 2021 [92]	Saudi Arabia	Middle East	322	171	21.9	116 (36.6)	44 (13.7)	NA	NA	GAD-7	NA	3/2020, two weeks period
											(5, 10)		
17	Bilgi et al., 2021 [107]	Turkey	Asia	178	127	21	132 (74.2)	66 (37.1)	NA	104 (58.4)	GAD-7	PHQ-9	6/1/2020 ~ 6/18/2020
						(median)					(5, 10)	(-, 10)	
18	Biswas et al., 2022 [130]	Bangladesh	Asia	425	265	NA	NA	NA	341 (80.2)	207 (48.7)	NA	PHQ-9	4/21/2020 ~ 5/10/2020
												(5, 10)	
19	Bolatov et al., 2021 [150]	Kazakhstan	Asia	798	NA	NA	340 (42.6)	124 (15.5)	475 (59.5)	220 (27.6)	GAD-7	PHQ-15	4/13/2020 ~ 4/19/2020
											(5, 10)	(5, 15)	
20	Cao et al., 2020 [29]	China	Asia	7143	4975	NA	1776 (24.9)	258 (3.6)	NA	NA	GAD-7	NA	NA
											(5, 10)		
21	Capdevila-Gaudens et al., 2021 [139]	Spain	Europe	5216	3979	21.41	2139 (41.0)	NA	1288 (24.7)	NA	STAI	BDI-II	2/17/2020 ~ 3/5/2020
											(31, 80)	(13, 63)	
22	Carletto et al., 2022 [147]	Italy	Europe	1329	869	NA	635 (47.8)	309 (23.3)	692 (52.1)	401 (30.2)	GAD-7	BDI-II	12/2020 ~ 2/2021
											(5, 10)	(13, 63)	
23	Chakeeyanun et al., 2023 [127]	Thailand	Asia	437	188	21.4	NA	NA	118 (27.0)	NA	NA	PHQ-9	1/2022
												(5, 10)	
24	Chang et al., 2021 [30]	China	Asia	4115	2489	20.2	1352 (32.9)	1079 (26.2)	1312 (31.9)	855 (20.8)	DASS-21	DASS-21	6/1/2020 ~ 6/15/2020
											(8, 10)	(10, 14)	
25	Chaudhuri et al., 2020 [57]	India	Asia	392	130	NA	129 (32.9)	90 (23.0)	176 (44.9)	117 (29.8)	DASS-21	DASS-21	5/2020
											(8, 10)	(10, 14)	
26	Chootong et al., 2022 [128]	Thailand	Asia	325	186	21	NA	42 (12.9)	199 (61.2)	101 (31.1)	GAD-7	PHQ-9	9/2021 ~ 10/2021
											(5, 10)	(5, 10)	
27	Christophers et al., 2021 [27]	United States	North America	1139	830	NA	583 (57.7)	198 (19.6)	620 (61.2)	249 (24.6)	GAD-7	PHQ-8	6/17/2020 ~ 7/17/2020
											(5, 10)	(5, 10)	
28	Çimen et al., 2022 [108]	Turkey	Middle East	2778	1869	20.69	NA	1235 (44.5)	NA	2442 (90.2)	GAD-7	PHQ-9	NA
											(5, 10)	(5, 10)	
29	Cinar Tanriverdi et al., 2023 [109]	Turkey	Middle East	904	488	21.3	636 (70.4)	460 (50.9)	587 (64.9)	409 (45.2)	DASS-21	DASS-21	6/2/2020 ~ 6/10/2020
											(8, 10)	(10, 14)	
30	de Souza et al., 2021 [112]	Brazil	South America	272	215	21	NA	137 (50.4)	NA	140 (51.5)	BAI	BDI-II	9/2020 ~ 2/2021
											(13, 63)	(13, 63)	
31	Deb N., Roy P., 2022 [58]	India	Asia	258	162	NA	183 (70.9)	159 (61.6)	210 (81.4)	201 (77.9)	DASS-21	DASS-21	7/2021 ~ 10/2021
											(8, 10)	(10, 14)	
32	Deng et al., 2021 [157]	China	Asia	1837	1227	21 (median)	749 (40.7)	167 (9.1)	791 (43.1)	212 (11.5)	GAD-7	PHQ-9	2/2021 ~ 4/2020
											(5, 10)	(5, 10)	
33	Ecker et al., 2022 [95]	United States	North America	212	148	NA	NA	NA	103 (48.6)	NA	NA	CESD-10	9/1/2020 ~ 12/31/2020
												(0, 10)	
34	Eid et al., 2021 [93]	Saudi Arabia	Middle East	336	NA	NA	161 (47.9)	72 (21.4)	193 (57.4)	79 (23.5)	DASS-21	DASS-21	5/2020 ~ 12/2020
											(10, 15)	(13, 21)	
35	Eleftheriou et al., 2021 [144]	Greece	Europe	559	389	NA	337 (67.4)	NA	415 (74.2)	NA	GAD-7	NA	4/22/2021 ~ 5/31/2021
											(-, 10)		
36–1	Ertek et al., 2022 [110]	Turkey	Middle East	1306	774	NA	NA	NA	838 (64.2)	NA	NA	CES-D	2019
												(16, -)	
36–2	Ertek et al., 2022 [110]	Turkey	Middle East	898	599	NA	NA	NA	816 (90.9)	NA	NA	CES-D	2021
												(16, -)	
37	Esmat et al., 2021 [131]	Egypt	Africa	238	147	22.2	NA	NA	187 (78.6)	132 (55.5)	NA	BDI-II	4/11/2020 ~ 5/3/2020
												(13, 63)	
38	Essadek et al., 2022 [102]	France	Europe	668	500	21.1	259 (38.7)	NA	255 (38.1)	NA	GAD-7	PHQ-9	4/27/2020 ~ 4/30/2020
											(5, 10)	(5, 10)	
39	Essangri et al., 2021 [135]	Morocco	Africa	549	406	22	342 (62.3)	141 (25.7)	410 (74.7)	251 (45.7)	GAD-7	PHQ-9	4/8/2020 ~ 4/18/2020
											(5, 10)	(5, 10)	
40	Frajerman et al., 2022 [103]	France	Europe	1128	817	NA	NA	658 (58.3)	NA	257 (22.8)	HAD	HAD	5/11/2021 ~ 6/13/2021
											(-, 10)	(-, 10)	
41–1	Gao et al., 2021 [32]	China	Asia	387	276	NA	95 (24.5)	74 (23.5)	51 (13.1)	18 (4.7)	DASS-21	DASS-21	6/23/2020 ~ 7/19/2020
											(8, 10)	(10, 14)	
41–2	Gao et al., 2021 [32]	China	Asia	315	225	NA	107 (27.6)	81 (25.7)	66 (21.0)	42 (13.3)	DASS-21	DASS-21	10/9/2020 ~ 10/11/2020
											(8, 10)	(10, 14)	
42	Gómez-Durán et al., 2022 [140]	Spain	Europe	173	136	22.5	117 (67.6)	60 (34.7)	74 (57.2)	46 (26.6)	GAD-7	PHQ-9	12/2021 ~ 3/2022
											(5, 10)	(8, 11)	
43	Guo et al., 2021 [96]	United States	North America	852	NA	NA	563 (66.1)	265 (31.1)	NA	NA	GAD-7	NA	6/2020 ~ 8/2020
											(5, 10)		
44	Guo et al., 2022 [33]	China	Asia	2048	1226	NA	606 (29.5)	NA	1076 (52.5)	NA	GAD-7	PHQ-9	7/8/2020 ~ 7/16/2020
											(5, 10)	(5, 10)	
45	Gupta et al., 2022 [59]	India	South Asia	118	46	NA	61 (51.6)	44 (37.3)	46 (39.0)	27 (22.9)	DASS-21	DASS-21	NA
											(8, 10)	(10, 14)	
46	Guse et al., 2021 [143]	Germany	Europe	887	562	NA	164 (18.5)	NA	183 (20.6)	NA	PHQ-4	PHQ-4	5/28/2020 ~ 6/7/2020
											(3, 6)	(3, 6)	
47	Halperin et al., 2021 [16]	United States	North America	1428	952	22.3	941 (65.9)	437 (30.6)	799 (56.0)	347 (24.3)	GAD-7	PHQ-9	4/13/2020 ~ 4/28/2020
											(5, 10)	(5, 10)	
48	Hassnain et al., 2021 [75]	Pakistan	Asia	230	130	NA	120 (52.2)	108 (47.0)	144 (62.6)	108 (47.0)	DASS-21	DASS-21	NA
											(8, 10)	(10, 14)	
49	Hjiej et al., 2022 [136]	Morocco	Africa	3174	2077	20.4	1437 (45.2)	NA	2063 (64.9)	NA	HADS	HADS	4/19/2020 ~ 4/23/2020
											(8, -)	(8, -)	
50	Huarcaya-Victoria et al., 2021 [120]	Peru	South America	1238	848	21.4	710 (57.4)	235 (19.0)	911 (73.4)	421 (34.0)	GAD-7	PHQ-9	4/24/2020 ~ 5/10/2020
											(5, 10)	(5, 10)	
51	Ismail et al., 2021 [116]	Malaysia	Asia	237	165	NA	46 (19.4)	NA	NA	NA	DASS-21	NA	11/12/2020 ~ 12/10/2020
											(8, 10)		
52	Jindal et al., 2020 [60]	India	Asia	432	NA	NA	NA	64 (14.8)	NA	NA	GAD-7	NA	5/13/2020 ~ 5/24/2020
											(-, 10)		
53	Junaid Tahir et al., 2022 [76]	Pakistan	Asia	261	NA	NA	11 (4.2)	4 (1.5)	47 (18.0)	7 (2.7)	SAS	SDS	3/30/2020 ~ 4/27/2020
											(50, 60)	(50, 60)	
54	Jupina et al., 2022 [97]	United States	North America	960	575	NA	388 (40.4)	NA	241 (25.1)	NA	GAD-2	PHQ-4	12/14/2020 ~ 1/10/2021
											(3, -)	(3, -)	
55	Kamran et al., 2022 [77]	Pakistan	Asia	324	223	NA	244 (75.3)	144 (59.0)	NA	NA	GAD-7	NA	6/1/2021 ~ 11/10/2021
											(5, 10)		
56	Khidri et al., 2022 [78]	Pakistan	Asia	864	366	NA	NA	NA	820 (94.9)	596 (69.0)	NA	PHQ-9	6/2020 ~ 8/2020
												(5, 10)	
57	Kim et al., 2022 [133]	South Korea	Asia	318	NA	NA	26 (8.17)	NA	47 (14.8)	NA	HADS	HADS	4/20/2020 ~ 5/1/2020
											(8, -)	(8, -)	
58	Kuman Tunçel et al., 2021 [111]	Turkey	Middle East	3105	1762	22.4	1563 (50)	719 (23.2)	NA	NA	BAI	NA	4/6/2020 ~ 5/7/2020
											(8, 16)		
59	Kumar et al., 2021 [79]	Pakistan	Asia	369	NA	NA	280 (75.9)	154 (41.7)	327 (88.6)	214 (58.0)	GAD-7	PHQ-9	7/2020 ~ 12/2020
											(5, 10)	(5, 10)	
60	Lee et al., 2021 [98]	United States	North America	687	443	NA	404 (58.8)	175 (25.5)	NA	NA	GAD-7	NA	4/20/2020 ~ 5/25/2020
											(5, 10)		
61	Leroy et al., 2021 [104]	France	Europe	4193	NA	NA	1048 (25.0)	NA	570 (13.6)	NA	STAI	BDI-II	4/17/2020 ~ 5/4/2020
											(-, 55)	(13, 63)	
62	Liu et al., 2021 [34]	China	Asia	131	NA	NA	65 (49.2)	NA	NA	NA	STAI-6	NA	4/2020 ~ 8/2020
											(53, -)		
63	Liu et al., 2020 [35]	China	Asia	217	127	21.7	48 (22.1)	16 (7.4)	77 (35.5)	24 (11.1)	GAD-7	PHQ-9	2/23/2020 ~ 4/2/2020
											(5, 10)	(5, 10)	
64	Lu et al., 2022 [36]	China	Asia	519	243	NA	NA	NA	78 (15.0)	27 (5.2)	NA	PHQ-9	11/2020
												(5, 10)	
65	Madaan et al., 2022 [61]	India	Asia	538	NA	NA	164 (30.5)	105 (19.5)	219 (40.7)	157 (29.2)	DASS-21	DASS-21	5/22/2020 ~ 6/5/2020
											(8, 10)	(10, 14)	
66	Manjareeka M., Pathak M., 2021 [62]	India	Asia	101	64	19.7	78 (77.2)	NA	NA	NA	STAI-6	NA	2/2020
											(40, -)		
67	Maroof et al., 2022 [80]	Pakistan	Asia	122	69	21.6	46 (37.7)	NA	NA	NA	GAD-7	NA	10/1/2020 ~ 11/15/2020
											(5, 10)		
68	Mendes et al., 2021 [113]	Brazil	South America	218	169	NA	110 (50.5)	NA	110 (50.5)	NA	BAI	BDI-II	9/21/2020 ~ 11/5/2020
											(8, 16)	(13, 63)	
69	Meng et al., 2021 [37]	China	Asia	1624	NA	NA	82 (5.1)	NA	121 (7.5)	NA	GAD-7	PHQ-9	2/14/2020 ~ 2/21/2020
											(5, 10)	(5, 10)	
70	Mishra et al., 2023 [63]	India	Asia	302	NA	NA	82 (27.2)	NA	93 (30.8)	NA	DASS-21	DASS-21	9/2020 ~ 10/2021
											(-, -)	(-, -)	
71	Mishra et al., 2022 [64]	India	Asia	284	169	20.6	80 (28.2)	NA	90 (31.7)	NA	DASS-21	DASS-21	10/2020 ~ 11/2021
											(-, -)	(-, -)	
72	Mohamed et al., 2022 [153]	Saudi Arabia	Middle East	1058	604	NA	585 (55.3)	463 (43.8)	793 (75.0)	641 (60.6)	DASS-21	DASS-21	4/1/2020 ~ 7/5/2020
											(8, -)	(10, -)	
73	Muhammad Alfareed Zafar et al., 2020 [153]	Pakistan	Asia	323	NA	NA	14 (4.3)	5 (1.5)	57 (17.6)	8 (2.5)	SAS	SDS	3/27/2020 ~ 4/22/2020
											(50, 60)	(50, 60)	
74	Nakhostin-Ansari et al., 2020 [145]	Iran	Middle East	323	169	23.7	123 (38)	46 (14.2)	89 (27.6)	35 (10.8)	BAI	BDI-II	4/8/2020 ~ 4/18/2020
											(10, 19)	(14, 20)	
75	Natalia D., Syakurah R.A., 2021 [124]	Indonesia	Asia	1027	NA	NA	491 (47.8)	NA	191 (18.6)	NA	DASS-21	DASS-21	7/14/2020 ~ 7/21/2020
											(8, -)	(10, -)	
76	Nguyen et al., 2022 [156]	Vietnam	Asia	5765	2726	21.7	467 (8.1)	NA	704 (12.2)	NA	GAD-8	PHQ-9	4/7/2020 ~ 5/31/2020
											(8, -)	(10, -)	
77	Ni et al., 2021 [38]	China	Asia	157	NA	NA	6 (3.8)	NA	76 (48.4)	2 (1.2)	SAS	SDS	3/4/2020
											(50,-)	(53, -)	
78	Nihmath Nisha et al., 2020 [65]	India	Asia	359	178	NA	271 (75.5)	149 (41.5)	268 (74.6)	160 (44.6)	GAD-7	CES-D	4/7/2020 ~ 6/7/2020
											(5, 10)	(5, 11)	
79	Nishimura et al., 2021 [148]	Japan	Asia	473	161	22	NA	34 (7.2)	NA	75 (15.9)	GAD-7	PHQ-9	6/8/2020 ~ 6/14/2020
											(-, 10)	(-, 10)	
80	Nugraha et al., 2023 [125]	Indonesia	Asia	718	555	20	468 (65.2)	NA	326 (45.4)	NA	DASS-21	DASS-21	8/31/2020 ~ 9/30/2020
											(7, 10)	(10, 13)	
81	Pattanaseri et al., 2022 [129]	Thailand	Asia	224	113	NA	NA	NA	80 (35.7)	NA	NA	PHQ-9	3/2020 ~ 10/2020
												(9, -)	
82	Paz et al., 2023 [99]	United States	North America	152	97	NA	102 (67.4)	56 (36.7)	101 (66.6)	38 (25.3)	GAD-7	PHQ-9	5/18/2021 ~ 6/4/2021
											(5, 10)	(5, 10)	
83	Pedraz-Petrozzi et al., 2021 [121]	Peru	South America	125	68	NA	74 (59.2)	16 (12.8)	92 (73.6)	43 (34.4)	GAD-7	PHQ-9	8/20/2020 ~ 11/20/2020
											(5, 10)	(5, 10)	
84	Pelaccia et al., 2021 [105]	France	Europe	1165	760	22.8	264 (22.7)	86 (7.4)	NA	NA	STAI-A	NA	5/7/2020 ~ 5/17/2020
											(55, 65)		
85	Peng et al., 2022 [23]	China	Asia	740	561	25 (median)	NA	164(22.2)	NA	250(33.8)	GAD-7	PHQ-10	10/2/2020 ~ 4/5/2021
											(-, 10)	(-, 10)	
86	Perissotto et al., 2021 [114]	Brazil	South America	347	229	22.6	206 (55.2)	NA	125 (33.5)	NA	HADS	HADS	3/2020 ~ 6/2020
											(9, -)	(9, -)	
87	Poon et al., 2021 [142]	Multi-countries	Asia	374	NA	NA	NA	114 (30.4)	NA	141 (37.7)	GAD-7	PHQ-9	5/2020
											(-, 10)	(-, 10)	
88	Ravikumar et al., 2022 [66]	India	Asia	221	115	NA	127 (57.4)	54 (24.4)	141 (63.8)	70 (31.7)	GAD-7	PHQ-9	1/2021 ~ 2/2021
											(5, 10)	(5, 10)	
89	Reddy C.R.E.T., Tekulapally K., 2022 [67]	India	Asia	164	NA	20.3	105 (68.6)	33 (21.6)	NA	NA	GAD-7	NA	12/2020
											(5, 10)		
90	Rehman et al., 2022 [82]	Pakistan	Asia	165	165	20.33	NA	106 (64.2)	NA	NA	VAS	NA	10/23/2020 ~ 11/30/2020
91	Risal et al., 2020 [138]	Nepal	Asia	416	176	22.2	NA	88 (21.2)	NA	62 (14.9)	HADS	HADS	NA
											(-, 11)	(-, 11)	
92	Rolland et al., 2022 [106]	France	Europe	7952	5710	NA	4557 (57.3)	NA	1589 (20.0)	NA	HAD	HAD	5/27/2021 ~ 6/27/2021
											(-,11)	(-,11)	
93	Rutkowska et al., 2021 [152]	Poland	Europe	3051	1773	22.3	NA	NA	1433 (47.0)	831 (27.2)	NA	BDI-II	3/2021 ~ 4/2021
												(-, 11)	
94	Saali et al., 2022 [100]	United States	North America	108	50	25.4	66 (61.1)	35 (32.4)	52 (48.1)	26 (24.1)	GAD-7	PHQ-8	6/2020 ~ 7/2020
											(5, 10)	(5, 10)	
95	Saddik et al., 2020 [155]	United Arab Emirates	Middle East	719	NA	NA	175 (24.3)	80 (11.1)	NA	NA	GAD-7	NA	3/11/2020 ~ 3/21/2020
											(5, 10)		
96	Saeed N., Javed N., 2021 [83]	Pakistan	Asia	234	111	20.7	225 (96.1)	155 (66.2)	200 (85.5)	151 (64.5)	GAD-7	PHQ-9	6//2020 ~ 8/2020
											(5, 10)	(5, 10)	
97	Safa et al., 2021 [19]	Bangladeshi	Asia	425	265	22	280 (65.9)	164 (38.6)	213 (50.1)	99(23.3)	HADS	HADS	4/21/2020 ~ 5/10/2020
											(9, -)	(9, -)	
98	Saguem et al., 2022 [154]	Tunisia	North Africa	251	207	21	140 (55.8)	128 (51.0)	170 (67.7)	144 (57.4)	DASS-21	DASS-21	4/11/2020 ~ 5/3/2020
											(8, 10)	(10,14)	
99	Santander-Hernández et al., 2022 [122]	Peru	South America	370	229	NA	255 (69.0)	142 (38.4)	290 (99.6)	171 (46.2)	GAD-7	PHQ-9	7/2020 ~ 10/2020
											(5, 10)	(5, 10)	
100	Saravia-Bartra et al., 2020 [123]	Peru	South America	57	37	NA	43 (75.4)	13 (22.8)	NA	NA	GAD-7	NA	4/2020 ~ 8/2020
											(5, 10)		
101	Sartorão Filho et al., 2020 [115]	Brazil	South America	340	251	NA	287 (84.4)	157 (46.2)	305 (89.7)	219 (64.4)	GAD-7	PHQ-9	5/18/2020 ~ 5/19/2020
											(5, 10)	(5, 10)	
102	Selvamani et al., 2022 [68]	India	Asia	304	NA	20.5	149 (49.0)	131 (43.1)	164 (53.9)	141 (46.4)	DASS-21	DASS-21	4/2020 ~ 8/2020
											(8, 10)	(10, 14)	
103	Shailaja et al., 2020 [69]	India	Asia	530	304	20.6	110 (20.8)	79 (14.9)	123 (23.2)	83 (15.7)	DASS-21	DASS-21	4/23/2020 ~ 4/29/2020
											(-, -)	(-, -)	
104	Shreevastava et al., 2022 [70]	India	Asia	1208	632	NA	811 (67.1)	488 (40.4)	NA	NA	GAD-7	NA	8/15/2020 ~ 10/15/2020
											(5, 10)		
105	Soltan et al., 2021 [132]	Egypt	Africa	282	181	20.3	175 (62.1)	159 (56.4)	249 (80.3)	212 (75.2)	DASS-21	DASS-21	5/1/2020 ~ 6/30/2020
											(8, 10)	(10, 14)	
106	Song et al., 2022 [40]	China	Asia	435	NA	NA	30 (6.9)	10 (2.3)	69 (15.9)	20 (4.6)	SAS	SAS	2/17/2020 ~ 2/23/2020
											(50, 60)	(50, 60)	
107	Srivastava et al., 2021 [71]	India	Asia	97	51	19.2	55 (56.7)	24 (24.7)	NA	NA	GAD-7	NA	NA
											(5, 10)		
108–1	Stanislawski et al., 2023 [101]	United States	North America	92	42	26.1	56 (60.9)	28 (30.4)	43 (46.7)	20 (21.7)	GAD-7	PHQ-8	6/2020
											(5, 10)	(5, 10)	
108–2	Stanislawski et al., 2023 [101]	United States	North America	87	NA	NA	46 (52.9)	21 (24.1)	34 (39.0)	12 (13.8)	GAD-7	PHQ-8	10/2020
											(5, 10)	(5, 10)	
108–3	Stanislawski et al., 2023 [101]	United States	North America	80	NA	NA	49 (61.3)	24 (30.0)	41 (51.3)	13 (16.3)	GAD-7	PHQ-8	2/2021
											(5, 10)	(5, 10)	
108–4	Stanislawski et al., 2023 [101]	United States	North America	67	NA	NA	40 (59.7)	17 (25.4)	25 (37.3)	9 (13.4)	GAD-7	PHQ-8	6/2021
											(5, 10)	(5, 10)	
109	Sudi et al., 2022 [117]	Malaysia	Asia	196	141	NA	NA	NA	134 (68.4)	86 (4.9)	NA	PHQ-9	NA
												(-, 10)	
110	Tee et al., 2022 [118]	Malaysia	Asia	378	250	23.1	167 (44.2)	NA	NA	NA	DASS-21	NA	5/2020 ~ 7/2021
											(-, -)		
111	Teh et al., 2023 [119]	Malaysia	Asia	371	247	NA	246 (66.3)	137 (36.9)	NA	NA	GAD-7	NA	1/27/2022 ~ 5/27/2022
											(5, 10)		
112	Tejoyuwono et al., 2021 [126]	Indonesia	Asia	133	NA	NA	20 (15.0)	16 (12.0)	18 (13.5)	11 (8.3)	DASS-21	DASS-21	6/2020 ~ 11/2020
											(8, 10)	(10, 14)	
113	Vala et al., 2020 [72]	India	Asia	250	140	NA	43 (17.2)	27 (10.8)	39 (15.6)	22 (8.8)	DASS-21	DASS-21	NA
											(8, 10)	(10, 14)	
114	Wu et al., 2022 [41]	China	Asia	1336	700	NA	376 (27.5)	NA	368 (27.6)	NA	SAS	SDS	6/23/2021 ~ 6/25/2021
											(50, -)	(50, 1-)	
115	Xiang et al., 2022 [42]	China	Asia	1207	571	NA	NA	NA	945 (78.4)	NA	NA	CES-D-10	2/2020 ~ 6/2021
												(10, -)	
116	Xiao et al., 2020 [43]	China	Asia	933	654	NA	160 (17)	43 (4.6)	236 (25.0)	71 (7.6)	GAD-7	PHQ-9	2/4/2020 ~ 2/12/2020
											(5, 10)	(5, 10)	
117	Xie et al., 2021 [44]	China	Asia	1026	653	NA	NA	NA	NA	230 (22.4)	NA	SDS	2/18/2020 ~ 2/22/2020
												(22 -)	
118	Xiong et al., 2021 [45]	China	Asia	382	256	21.3	NA	58 (15.2)	NA	41 (10.7)	DASS-21	DASS-21	2/20/2020 ~ 3/20/2020
											(-, 10)	(-, 14)	
119	Yang Q. et al., 2022 [46]	China	Asia	3473	2388	19.7	NA	308 (8.8)	NA	623 (17.9)	GAD-7	PHQ-9	10/2020 ~ 4/2021
											(5, 10)	(5, 10)	
120	Yang X. et al., 2022 [47]	China	Asia	6226	3742	NA	1423 (22.9)	NA	2206 (35.4)	NA	GAD-7	PHQ-9	2/11/2020 ~ 2/19/2020
											(5, 10)	(5, 10)	
121	Yin et al., 2021 [48]	China	Asia	5982	3591	21.7	1365 (22.8)	246 (4.1)	2100 (35.1)	486 (8.1)	GAD-7	PHQ-9	2/11/2020 ~ 2/18/2020
											(5, 10)	(5, 10)	
122	Yuan et al., 2021 [49]	China	Asia	519	243	NA	148 (28.5)	NA	164 (31.6)	NA	GAD-7	PHQ-9	11/2020
											(5, -)	(5, -)	
123	Yun et al., 2021 [134]	South Korea	Asia	454	165	19.1	84 (18.5)	NA	54 (11.9)	NA	GAD-7	PHQ-9	6/2020 ~ 7/2020
											(-, 10)	(-, 10)	
124	Zhang K. et al., 2021 [50]	China	Asia	1041	545	21.3	211 (20.3)	181 (17.4)	279 (26.8)	199 (19.1)	DASS-21	DASS-21	4/2020
											(7, 20)	(9, 28)	
125	Zhang L. et al., 2021 [51]	China	Asia	142	74	NA	43 (30.3)	NA	58 (40.8)	NA	GAD-7	PHQ-9	3/2020
											(5, 10)	(5, 10)	
126	Zhang X. et al., 2021 [52]	China	Asia	563	NA	NA	20 (3.6)	NA	57 (10.1)	NA	SAS	SDS	2/21/2020 ~ 2/24/2020
											(50, -)	(53, -)	
127	Zhao et al., 2022 [53]	China	Asia	565	393	20.8	NA	NA	NA	102 (18.1)	NA	PHQ-9	5/2020 ~ 7/2020
												(-, 10)	
128	Zhao et al., 2021 [54]	China	Asia	666	404	20	NA	NA	NA	64 (9.6)	NA	PHQ-9	3/20/2020 ~ 4/10/2020
												(-, 10)	
129	Zheng et al., 2021 [55]	China	Asia	468	283	21.5	153 (32.7)	53 (11.3)	217 (46.4)	97 (20.7)	GAD-7	PHQ-9	12/17/2020 ~ 12/19/2020
											(5, 10)	(5, 10)	
130	Zhong et al., 2021 [56]	China	Asia	746	502	NA	NA	NA	242 (32.4)	NA	NA	SDS	4/2020 ~ 5/2020
												(53, -)	

*GAD-7* Generalized Anxiety Disorder-7, *BAI* Beck Anxiety Inventory, *STAI-6* State-Trait Anxiety Inventory-6, *PHQ-9* Patients Health Questionnaire-9, *SAS* Zung Self-rating Anxiety Scale, *SDS* Zung Self-rating Depression Scale, *BDI-II* Beck Depression Inventory-II, *HADS* Hospital Anxiety and Depression Scale, *DASS-21* Depression, Anxiety and Stress Scale-21, *CES-D* Center for Epidemiology Studies for Depression scale, *VAS* Visual Analogue Scale, *NA* Not Available

^a^Anxiety moderate and severe

^b^Depression moderate and severe

^c^(m, MS) = anxiety symptom cutoff value (mild, moderate and severe)

^d^(m, MS) = depressive symptom cutoff value (mild, moderate and severe)

### Risk of bias in studies

The JBI scale was used to appraise the quality of the 130 cross-sectional studies. The results of quality assessment are presented in Additional File 3: Appendix 3.

### Prevalence of anxiety and depression among medical students

#### Anxiety

**Fig. 2:**
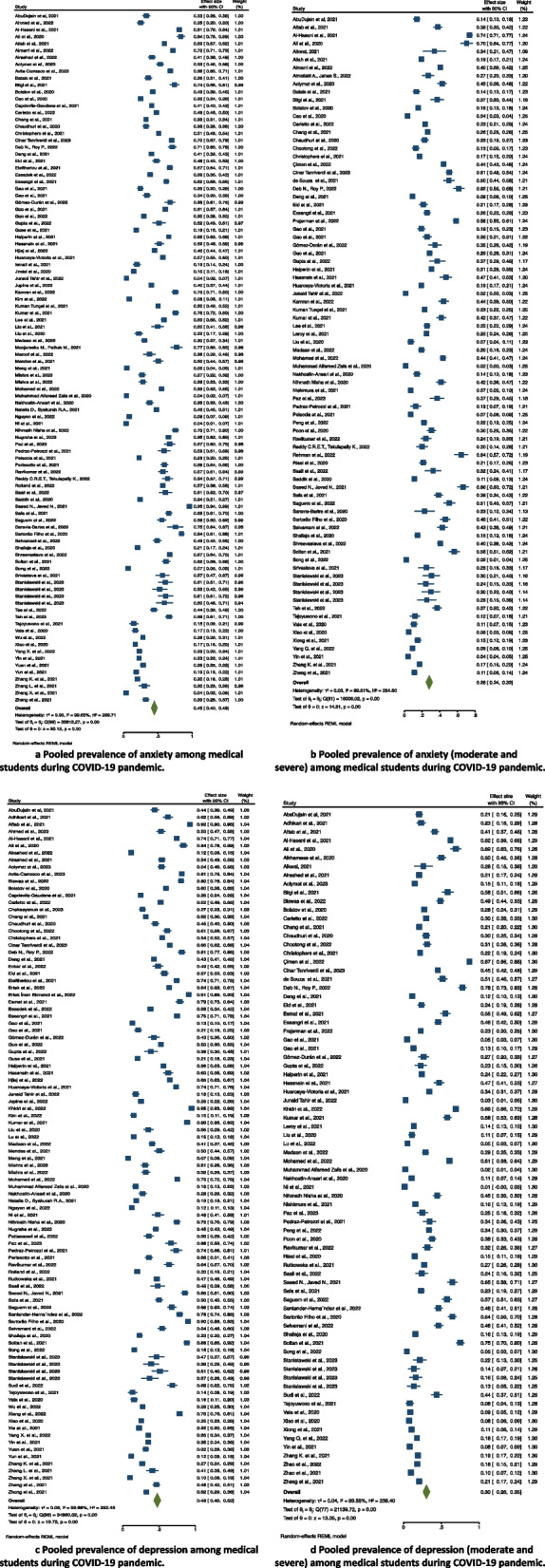
Pooled prevalence of anxiety and depression among medical students during COVID-19 pandemic. **a** Pooled prevalence of anxiety among medical students during COVID-19 pandemic. **b** Pooled prevalence of anxiety (moderate and severe) among medical students during COVID-19 pandemic

One hundred studies that included 41,620 participants reported anxiety using the Generalized Anxiety Disorder-7 (GAD-7), the Depression, Anxiety and Stress Scale (DASS), Beck Anxiety Inventory (BAI), Zung Self-rating Anxiety Scale (SAS), and Hospital Anxiety and Depression Scale (HADS), State-Trait Anxiety Inventory (STAI), and Patient Health Questionnaire (PHQ). The pooled prevalence of anxiety among medical students during the COVID-19 pandemic was 45% (95% confidence interval [CI], 40%–49%, *I2* = 99.65%, *p* < 0.001). The forest plot is displayed in Fig. 2a.

#### Anxiety MS (moderate and severe)

Eighty-two studies that included 17,495 participants reported anxiety MS using the GAD-7, DASS-21, BAI, STAI, Zung SAS, Visual Analogue Scale (VAS), and HADS. The pooled prevalence of anxiety MS among medical students during the COVID-19 pandemic was 28% (95% CI, 24%–32%, *I2* = 99.61%, *p* < 0.001). The forest plot is displayed in Fig. 2b.

#### Depression

Ninety-seven studies that included 35,828 medical students reported depression using the Patient Health Questionnaire-9 (PHQ-9), DASS-21, Zung Self-rating Depression Scale (SDS, Beck Depression Inventory-II (BDI-II), and HADS. The pooled prevalence of depression among medical students during the COVID-19 pandemic was 48% (95% CI, 43%–52%, *I2* = 99.66%, *p* < 0.001). The forest plot is displayed in Fig. 2c.

#### Depression MS (moderate and severe)

Seventy-eight studies that included 15,719 medical students reported depression MS using the PHQ-9, DASS-21, Zung SDS, BDI-II, HADS, and the Self-Rated Depression Scale. The pooled prevalence of depression MS among medical students during the COVID-19 pandemic was 30% (95% CI, 26%–35%, *I2* = 99.58%, *p* < 0.001). The forest plot is displayed in Fig. 2d.

There was high heterogeneity between studies, with I^2^ ranging from 99.58%–99.66%.

### Sensitivity analysis

**Fig. 3:**
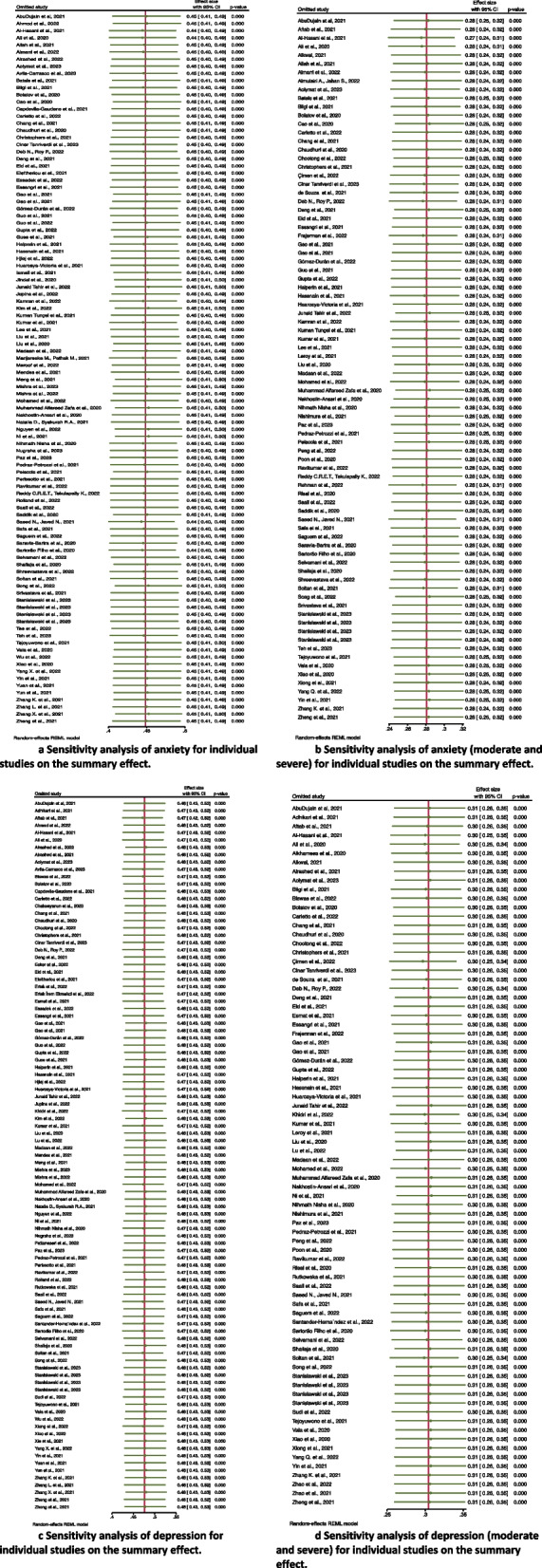
Sensitivity analysis of anxiety and depression for individual studies on the summary effect. **a** Sensitivity analysis of anxiety for individual studies on the summary effect. **b** Sensitivity analysis of anxiety (moderate and severe) for individual studies on the summary effect. **c** Sensitivity analysis of depression for individual studies on the summary effect. **d** Sensitivity analysis of depression (moderate and severe) for individual studies on the summary effect

We performed sensitivity analysis and confirmed the stability and reliability of the results. We used leave-one-out meta-analysis to identify influential studies. Figure 3a shows that the corresponding pooled prevalence of anxiety ranged from 44% to 45%. Figure 3b shows that the corresponding pooled prevalence of anxiety MS varied from 27% to 28%. Figure 3c shows that the corresponding pooled prevalence of depression ranged from 47% to 48%. Figure 3d shows that the corresponding pooled prevalence of depression MS varied from 30% to 31%. This was not substantially altered. The statistically similar results revealed that no single study influenced the stability of the overall prevalence estimates in the meta-analysis.

### Publication bias

**Fig. 4:**
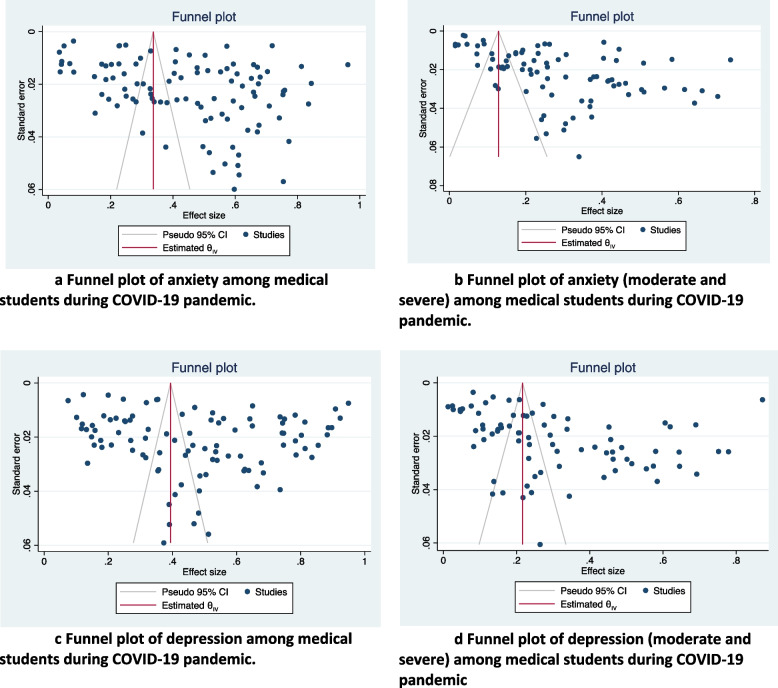
Funnel plot of anxiety and depression among medical students during COVID-19 pandemic. **a** Funnel plot of anxiety among medical students during COVID-19 pandemic. **b** Funnel plot of anxiety (moderate and severe) among medical students during COVID-19 pandemic. **c** Funnel plot of depression among medical students during COVID-19 pandemic. d Funnel plot of depression (moderate and severe) among medical students during COVID-19 pandemic

Visual inspection of the funnel plot revealed relative symmetry for anxiety and depression (Fig. 4a and c) and asymmetry for anxiety MS and depression MS (Fig4b and d). Begg’s (z = 1.25, p = 0.210) tests showed no potential risk of publication bias for the overall prevalence of anxiety. However, Egger’s (*z* = 3.63, p = 0.0003) tests showed a potential risk of publication bias for the overall prevalence of anxiety. Begg’s (z = 1.54, p = 0.124) tests showed no potential risk of publication bias for the overall prevalence of anxiety MS. However, Egger’s (*z* = 3.82, p = 0.0001) tests showed a potential risk of publication bias for the overall prevalence of anxiety MS. Both Begg’s (z = 0.60, p = 0.551) and Egger’s (*z* = 0.72, p = 0.469) tests showed no potential risk of publication bias for the overall prevalence of depression. Finally, both Begg’s (z = 3.56, p = 0.0004) and Egger’s (*z* = 2.68, p = 0.0073) tests showed a potential risk of publication bias for the overall prevalence of depression MS.

### Subgroup analysis

The results of subgroup analysis are presented in Table 2. In the evaluation according to continent or region, the pooled prevalence of anxiety was high in North and South America (60.7%, 95% CI, 56.0%–65.4%), intermediate in the Middle East and Africa (53.4%, 95% CI, 45.8%–61.0%) and in Europe (45.0%, 95% CI, 32.2–57.9), and low in Asia (37.3%, 95% CI, 31.1%–43.5%). The pooled prevalence of anxiety MS showed no statistical significance. The pooled prevalence of depression was high in the Middle East (61.1%, 95% CI, 50.3%–71.9%), intermediate in North and South America ( 56.5%, 95% CI, 47.7%–65.2%), and in Asia (41.7%, 95% CI, 35.4%–47.9%), and low in Europe (39.9%, 95% CI, 27.1%–52.7%). The pooled prevalence of depression MS was high in the Middle East (43.6%, 95% CI, 31.8%–55.4%), intermediate in North and South America (30.2%, 95% CI, 21.7%–38.7%), and in Asia (26.6%, 95% CI, 20.5%–32.7%), and low in Europe (23.9%, 95% CI, 18.1%–29.8%).

**Table 2:** Subgroup analyses of prevalence of anxiety and depression in medical students

Subgroups	Number of studies, n	Prevalence, % (95%CI)	*p-*value	Number of studies, n	Prevalence, % (95%CI)	*p-*value	Number of studies, n	Prevalence, % (95%CI)	*p-*value	Number of studies, n	Prevalence, % (95%CI)	*p-*value
		Anxiety			Anxiety MS^a^			Depression			Depression MS^b^	
Continent												
Asia	57	37.3 (31.1–43.5)	0.000	43	25.3 (19.7–30.9)	0.455	56	41.7 (35.4–47.9)	0.002	44	26.6 (20.5–32.7)	0.029
Middle East and Africa	17	53.4 (45.8–61.0)		18	33.3 (25.1–41.4)		15	61.1 (50.3–71.9)		15	43.6 (31.8–55.4)	
North and South America	18	60.7 (56.0–65.4)		15	28.8 (23.5–34.1)		17	56.5 (47.7–65.2)		13	30.2 (21.7–38.7)	
Europe	8	45.0 (32.2–57.9)		5	29.7 (13.2–46.2)		8	39.9 (27.1–52.7)		5	23.9 (18.1–29.8)	
Continent Asia												
Asia	57	37.3 (31.1–43.5)	0.000	43	25.3 (19.7–30.9)	0.125	56	41.7 (35.4–47.9)	0.004	44	26.6 (20.5–32.7)	0.059
Rest of the world	43	54.8 (50.2–59.4)		38	31.0 (26.2–35.9)		40	54.9 (48.5–61.3)		33	35.3 (28.5–42.1)	
Gender												
Female	30	46.1 (38.4–53.8)	0.086	17	22.9 (15.6–30.3)	0.478	26	46.1 (37.5–54.8)	0.136	15	29.1 (16.5–41.8)	0.644
Male	29	37.1 (30.3–43.9)		16	19.4 (13.1–25.7)		26	37.5 (30.0–44.9)		15	25.2 (14.4–36.0)	
Study year												
Pre-clinical	20	51.7 (41.8–61.6)	0.396	21	29.7 (21.2–38.2)	0.765	15	43.7 (29.5–57.9)	0.800	15	39.0 (26.0–52.0)	0.356
Clinical	16	45.4 (34.8–56.0)		17	22.2 (14.7–29.8)		10	41.3 (29.6–53.1)		13	30.3 (17.3–43.3)	

^a^Anxiety moderate and severe

^b^Depression moderate and severe

With categories divided into Asia and the rest of the world, the pooled prevalence of anxiety was lower in Asia than the rest of the world (37.3%, 95% CI, 31.1%–43.5% versus 54.8%, 95% CI, 50.2%–59.4%). The pooled prevalence of depression was lower in Asia than in the rest of the world (41.7%, 95% CI, 35.4%–47.9% versus 54.9%, 95% CI, 48.5%–61.3%). The pooled prevalence of anxiety MS and depression MS was lower in Asia than in the rest of the world, but without statistical significance.

When evaluating the effect of sex, the pooled prevalence of anxiety, anxiety MS, depression, and depression MS revealed higher trends in female than male. When evaluating medical students’ year of study, the pooled prevalence of anxiety, anxiety MS, depression, and depression MS revealed higher trends in the pre-clinical years than the clinical years. However, there was no significance regarding the effect of sex and medical students’ year of study.

### Meta-regression

We identified various levels of heterogeneity among studies and in subgroup analysis, and therefore meta-regression was used to explore potential sources of heterogeneity. As a potential source of heterogeneity, continent or region and survey date (the month during 2020 in which the last survey was conducted) were included in the meta-regression analysis. The results of meta-regression analysis are shown in Table 3. Continent was significantly associated with anxiety (coefficient 0.176; 95% CI, 0.094–0.257), and depression (coefficient 0.132; 95% CI, 0.040–0.224). Medical students in Asia showed a lower prevalence of anxiety and depression than those on other continents. The survey date (February to June, 2020) was significantly and positively correlated with anxiety (coefficient 0.109; 95% CI, 0.061–0.157), anxiety MS (coefficient 0.078; 95% CI, 0.038–0.117), depression (coefficient 0.079; 95% CI, 0.028–0.129), and depression MS (coefficient 0.083; 95% CI, 0.030–0.136). For every one-month increase, 10.9%, 7.8%, 7.9%, and 8.3% increase in the prevalence of anxiety, anxiety MS, depression, and depression MS, respectively. The survey date (May to December, 2020) was negatively correlated with anxiety, anxiety MS, depression, and depression MS. However, there were no statistical significance.

**Table 3:** Meta-regression for the prevalence of anxiety and depression in medical students

	Anxiety			Anxiety MS^a^			Depression			Depression MS^b^		
	Coefficient	95%CI	R squared	Coefficient	95%CI	R squared	Coefficient	95%CI	R squared	Coefficient	95%CI	R squared
Continent (reference group = Asia)	0.176**	0.094–0.257	14.7	0.058	-0.017–0.132	1.8	0.132**	0.040–0.224	6.9	0.088	-0.004–0.180	3.4
Survey date (February 2020 to June 2020)	0.109**	0.061–0.157	29.5	0.078**	0.038–0.117	27.5	0.079**	0.028–0.129	16.7	0.083**	0.030–0.136	18.8
Survey date (May 2020 to December 2020)	-0.010	-0.034–0.014	0.0	-0.005	-0.025–0.015	0.0	-0.010	-0.040–0.019	0.0	-0.018	-0.046–0.009	1.83

^a^Anxiety moderate and severe

^b^Depression moderate and severe

*p< 0.05

**p< 0.01

## Discussion

This systematic review and meta-analysis of 130 articles that included 132,068 participants revealed that the pooled estimates of prevalence showed that 45% of medical students have experienced anxiety and 48% have experienced depression. Furthermore, 28% of medical students have experienced anxiety MS and 30% of medical students have experienced depression MS. Moderate and severe anxiety and depression are of clinical importance as conditions for which further assistance and intervention might be needed. Our findings demonstrated that anxiety and depression are important issues for medical students during the COVID-19 pandemic, with wide variation in the prevalence among different studies.

To our knowledge, this is the most updated meta-analysis to simultaneously report anxiety and depression, as well as moderate and severe anxiety and depression, among medical students during the COVID-19 pandemic. We further investigated the characteristics of studies that that reported the influence on the prevalence of these conditions. The pooled prevalence indicated that continent was significantly associated with the prevalence of anxiety and depression in this population. Medical students’ sex and year of study (pre-clinical and clinical) were not significantly associated with the prevalence of anxiety and depression. Meta-regression analysis revealed that the date of survey was associated with mental health problems among medical students. From February to June in 2020, the prevalence of anxiety and depression among medical students increased over time. The increasing trend echoed the results of a previous study conducted among students of higher education, in which the authors believed that end-of-year examinations might account for this trend [157]. Moreover, we believe that the severity of the pandemic might have an influence on trends in anxiety and depression in this population. Further study is needed to confirm these results. These factors may have an impact on the learning of medical students and medical education.

### Impact of COVID-19 pandemic on medical education

Consequent to the unprecedented COVID-19 pandemic, pedagogic changes have resulted in a paradigm shift in teaching and learning processes. Medical education has been disrupted owing to the closure of medical schools or barring of patient contact during the pandemic. The traditional face-to-face teaching mode had largely been replaced by online learning [158–160]. Alternative modes of teaching and learning, including web-based learning, role play, video vignettes, and use of both live and mannequin-based simulated patients, have been used to minimize disruption to medical education [161]. Many challenges have arisen in this shift from traditional teaching methods to online learning. Study load and workload, enhanced engagement, and technical issues might affect student and faculty satisfaction [162]. One qualitative study provided recommendations emphasizing three important axes of institutional capacity, effective learning and assessment, and human resources, which would lead to planning and implementation of successful online learning activities [163].

### Associated stressors: personal, academic, environmental and cultural, and pandemic factors

The ongoing COVID-19 pandemic has had a great impact on medical students. The pooled prevalence of anxiety among medical students in our study was higher than the prevalence in a previous report [21]. One study found a 28% pooled prevalence of anxiety among medical students globally in September 2020 [21], which is much lower than our finding. A possible reason might be that half of the studies included in that report were conducted in China, and the sample sizes in those studies resulted in 89% of students being Chinese, which could limit generalization of the results. The pooled prevalence of depression among medical students in our study was higher than the prevalence in another study [22] reporting a 31% pooled prevalence of depression globally, but similar to the recent study [23] reporting a 38% pooled prevalence of anxiety and 41% pooled prevalence of depression among medical students. Notably, 28% of medical students reported anxiety MS and 30% reported depression MS. Medical institutes should make greater effort to identify those students in need of clinical intervention and provide timely assistance. Many reported stressors can induce symptoms of anxiety and depression in medical students, particularly during the COVID-19 pandemic. These can be categorized into several factors, including personal, academic, environmental and cultural, and pandemic factors.

#### Personal factors

In terms of personal factors, sex has been significantly associated with anxiety and depression among medical students in many studies, with most reporting that women have a higher prevalence of anxiety [16, 19, 43, 48, 65, 87, 98, 111, 114, 115, 120, 132, 135, 137, 145, 155] and depression [16, 19, 43, 48, 65, 69, 114, 115, 120, 131, 135]. Only one study reported that male students had a high risk of anxiety [32], and one study reported that male students had a high risk of depressive symptoms [164]. No difference in sex was reported for anxiety [20, 29, 35, 69, 107, 138] or for depression [20, 35, 107, 138] in numerous studies. Although our results revealed that sex was not significantly associated with anxiety or depression, women tended to have a higher prevalence of anxiety and depression. Having a history of psychiatric consultation or psychologic or mental problems was associated with anxiety and depression [132, 135, 138, 165]. Having negative thoughts or engaging in negative actions and feeling depressed was associated with a greater likelihood of anxiety [43]. Individuals who engage in negative thinking or actions, and those with anxiety levels have greater odds of experiencing some level of depression [43]. Maintaining a healthy lifestyle has been associated with less depression symptoms [43, 52].

#### Academic factors

Regarding academic factors, the influence of medical students’ year of study on anxiety and depression remains controversial. Although our results revealed that the pre-clinical and clinical years of study were not significantly associated with anxiety or depression, students in the pre-clinical years tended to have a higher prevalence of anxiety and depression than those in clinical years. Some studies have reported that students in the pre-clinical years of study have a higher prevalence of anxiety [16, 48, 92, 114, 120, 137] and depression [16, 48, 114, 120, 135, 137]. One study reported no significant differences in anxiety and depression between pre-clinical and clinical groups [138]. However, a past study found that students in their clinical phase of study had greater anxiety levels [155]. Medical students in clinical training, especially those participating in higher-risk unit rotations, have a greater risk of exposure to infectious diseases, which has a considerable impact on their mental health [155]. Further research is needed to confirm these results.

Some studies have reported that worrying about academic delays was a stressor leading to higher levels of anxiety [29, 120, 166] and depression [120]. One study reported that students with a higher grade point average (GPA) experienced less anxiety and depression [145]. A study in Japan found that the sudden shift to online education was associated with greater odds of having generalized anxiety and being depressed [148]. However, one study reported the mental health of medical students improved after the transition from traditional to online learning during the quarantine period [150]. In that study, 65.2% of those concerned about the shift to online education felt that this learning mode was less effective than in-person education [148]. Previous studies have reported the utility and equivalent effectiveness of online learning in comparison with offline or in-person learning [167]. Medical students who said that they would request food assistance and mental health care resources from the university in the case of future COVID-19 outbreaks also had greater odds of having generalized anxiety and being depressed [148]. Being unable to fully concentrate on their studies during the COVID-19 pandemic was found to be associated with a significantly higher risk of anxiety among medical students [19]. One study compared undergraduate and graduate students and found that graduate students had a higher risk of anxiety [43]. The authors explained that this may be owing to increased pressure regarding job-seeking or completion of a thesis required for graduation.

#### Environmental and cultural factors

Worrying about the economic effects of the pandemic and influence of the pandemic on daily life was found to be related to anxiety levels [29]. Living in an urban area, living with parents, and family financial stability have been identified as protective factors against anxiety [29, 120] and depressive symptoms [120]. One study reported that financial hardship during periods of lockdown and social distancing were associated with anxiety and depression [115]. However, another study reported that family financial stability was not associated with anxiety and depressive symptoms [20]. Students living alone had a higher prevalence of anxiety [20, 89]. A study showed that quarantine at home with family was not associated with anxiety and depression [138]. Another study showed that living away from the family during the pandemic had no association with depression or anxiety [69]. Having accurate knowledge was found to be a protective factor against anxiety [32] and depression [32, 81]. One study reported that a lower anxiety level might be associated with greater exposure to mass media and social media and stressed the importance of information during a pandemic [168]. Fear of being assaulted or insulted on the way to the hospital or at home was related to a significantly greater risk of anxiety among medical students [19]. Studies reported the strongly significant association of perceived social support with reduced levels of anxiety and depression [48, 165, 169], as well as improved quality of life [169]. Thus, provision of effective social support is paramount to lowering psychological stress during a global crisis [19].

#### Pandemic factors

Finally, pandemic factors such as social distancing and isolation from family members might worsen anxiety disorders [20, 166]. Having relatives or acquaintances with COVID-19 infection is a risk factor for increased anxiety [16, 29]. However, one study reported that having family members or friends who were infected with COVID-19 was not statistically associated with anxiety and depressive symptoms [20]. Possible COVID-19 exposure or having contact with patients who have COVID-19 infection is associated with anxiety [138, 155] and depression [138], as is experiencing COVID-19 symptoms [145]. Living in a location with a high prevalence of COVID-19 infections was found to be associated with a higher prevalence of severe anxiety and depression [48, 135]. One study compared the prevalence of anxiety disorders between students at two universities in China and found that the prevalence was significantly higher in Wuhan, which was far more severely affected by COVID-19 than Beijing [43]. More than 25 days of confinement was associated with severe anxiety and depressive symptoms [135]. One study found that most students reported being worried about transmitting COVID-19 to a family member or friends, and 65% of them worried about catching the virus themselves [155]. Another study reported that transmitting the virus to family members was not associated with anxiety or depression [19]. Students who were highly or moderately concerned about becoming infected were at higher risk (3.5-fold, 1.5-fold, respectively) of anxiety, as compared with students with no concerns regarding contracting COVID-19 infection [19]. Students who were highly or moderately concerned about contracting COVID-19 infection were also at higher risk (2.75-fold, 1.96-fold, respectively) of having depressive symptoms compared with their counterparts who were unconcerned [19]. One study reported that students with a low degree of concern about COVID-19 had a high risk of depressive symptoms [164].

### Other factors impact on anxiety and depression of medical students

When organized geographically, continent was also significantly associated with mental health problems among medical students in our study. Students in North and South America and Africa had the highest prevalence of anxiety whereas those in Asia had a lower prevalence of depression and depression MS. These results might be explained by the severity of the pandemic in different countries and regions. The COVID-19 pandemic has been more severe on the North and South American continents than on other continents, with the highest number of confirmed cases and deaths in the United States [2]. Another reason might be cultural influences. Mental disorders are considered a social stigma in many countries, especially Asian countries. Self-report measures of mental disorders might not reflect the real situation among medical students in Asian countries, which might explain the difference in the prevalence of anxiety and depression among different continents.

Studies comparing medical and non-medical students found that non-medical students had higher anxiety levels [45, 155] and depression [45, 55] than medical students. Possible reasons for these findings might be that medical students have better knowledge of the virus that causes COVID-19, the disease prognosis, and transmission and control measures. Our finding is in line with those of other studies stressing the importance of information during a pandemic [168]. In a comparison of medical and dental students, dental students reported higher levels of anxiety [155] as well as higher levels of anxiety and depression [170, 171]. This might be because dental students are in very close proximity to patients when providing dental care, which may increase the likelihood of exposure to highly transmissible respiratory viruses, thereby increasing the potential risk of COVID-19 infection owing to the nature of the dental field itself [172].

### Implications and recommendations

Medical students have been identified as having a high risk of developing mental health problems [17]. Amid the COVID-19 crisis, some medical students might be in a challenging position. To limit the use of personal protective equipment and to ensure the safety of medical students, clerkships and clinical activities have been suspended by some medical schools [173]. In contrast, medical students in some regions have been urged to participate in patient care, sometimes even earning credit toward their degree under certain circumstances [174]. According to one study, higher rates of depression, suicidal ideation, and stigmatization around depression have been noted in these students [175]. Depressed medical students might feel less respected because their coping skills may be viewed as inadequate or they may be considered less able to handle their responsibilities. Therefore, these individuals might feel that seeking mental health counseling services for depression is risky, making them less likely to seek support or treatment for depression [175]. It is important to safeguard the mental health of medical students with an effective plan to support their wellness as well as their education. Interventions targeting the many predisposing psychological factors in COVID-19-related anxiety and depression symptoms among medical students should be adopted by medical college administrators and policy makers to ameliorate psychological distress, which may negatively impact students’ academic performance [101, 176]. Several strategies had been investigated [177, 178, 179, 180]. Implications and recommendations for different levels, including medical students, medical schools and institutions, and policymakers, are presented in Table 4.

**Table 4:** Implications and recommendations

Levels	Description
Medical students	• Encouraging vaccination and good health habits (hand hygiene, wearing masks, and regular exercise) will provide them with adequate protection and help to lower anxiety levels and lessen depressive symptoms • Encouraging medical students to obtain accurate information regarding the pandemic and to live with their family or maintain active contact with family members, classmates, and faculty will help decrease their mental health burden • Actively seeking help should be recommended, especially for those who are feeling symptoms of anxiety and depression, and students with a history of mental health problems should seek professional mental health care [16] • Encouraging a healthy lifestyle and cultivating resilience will be beneficial for medical students’ mental health
Medical schools and institutions	• Provide timely and accurate information and education regarding the pandemic to medical students • Medical college administrators should ensure an optimal alternative learning environment for every medical student to continue their education • Schools and institutions should devote greater resources to building effective distance learning platforms and online courses as well as development programs for faculty to teach these courses • Virtual teaching programs can help medical students build their clinical competence during the pandemic • Schools and institutions can supply students with resources, including counseling, peer advocacy, and social support [29, 48, 166]. Schools and institutions should make regular connections with medical students, and mentors should contact their students regularly and help them if needed, especially those at higher risk of anxiety or depression, such as women and students with a lower GPA or a history of mental health problems • Medical schools and institutions should screen medical students to assess their mental health status and provide counseling and referrals to professionals if risks are identified. Screening university students on a regular basis can help faculty to identify highly anxious students early and guide them to receive help via targeted interventions that promote psychological well-being or services like pastoral counseling, mental health support, and instruction in coping mechanisms [177] • Mental health interventions should be included in the crisis response and should aim to destigmatize psychological problems, encourage communication, and provide psychological support [135] • Schools and institutions should identify those students who have a heavy financial burden and provide essential support to alleviate this potential stressor • Medical schools and institutions should provide mindfulness training or resilience-development programs for medical students to help them relieve stress. Additionally, mindfulness-based therapy [178] and internet-based cognitive behavioral therapy (I-CBT) can be useful to treat insomnia and stress among medical students [179, 180]. There is also a global need to implement strategies to build coping skills and resilience in a crisis among medical students and to take measures to prepare them for a disaster [111] • Clinical units should encourage students in clinical training to receive vaccines and maintain good health habits and should provide necessary protective equipment and arrange appropriate clinical training environments to decrease the risk of infection among medical students
Policymakers	• Policymakers should provide sufficient vaccines for medical students as a priority group. Adequate personal protective equipment should also be provided for health care workers and medical students • Measures must be taken to make the confinement period as short as possible • Authorities should implement policies to promote and build distance learning platforms as well as resilience-development programs. Disaster medicine training and curricula should be used globally to improve students’ knowledge, attitudes, and skills • Medical students can certainly contribute to the community, health care system, and society; however, mobilization of medical students to help in the COVID-19 response must be voluntary • The provision of effective social support is critical during a pandemic. Presenting effective examples from other countries might help medical students to overcome psychological distress associated with the COVID-19 pandemic

#### Study strengths and limitations

Our study has several strengths. The search strategy was comprehensive. As far as we know, our study was the most updated analysis and comprehensive review on this topic. Moreover, our implications and recommendations would be helpful for medical students, schools and institutions, and policymakers developing strategies to promote mental health for medical students when encountering the pandemic. Our review also had several limitations. Firstly, although the identified articles involved a large number of participants, meta-analysis and quantitative analysis revealed a variety of heterogeneity in the data. Some studies have also reported high heterogeneity in anxiety and depression among medical students [21, 22]. Publication bias is possible, especially because more than half of studies were conducted in Asia.

Secondly, the search strategies influenced the articles searched as well as the number of articles retrieved. For instance, the use of wildcards, adjacencies, and truncations in a keyword search might identify more relevant studies. Moreover, including the outcome measures (sometimes depression or anxiety was a secondary outcome and might not be discussed in the abstract or title) or using more or different key terms (such as “mental health” or “mental wellness”) in the search strategy might have an influence on the search results. Therefore, the possibility remains that some unidentified or unpublished articles and gray literature were not included in our study. Moreover, because the search was limited to English language, studies in other languages were not included. Furthermore, medical students were limited to M.D. and M.B.B.S. programs, so some different programs for medical students might not be included in the study.

Thirdly, many other factors that might have an influence on heterogeneity of the data, such as family history, family income, emotional trauma, residential area, smoking, and substance use, were not included in this study. Although our results revealed the prevalence of anxiety and depression among medical students in some countries and regions, data were lacking for other countries and regions.

Fourthly, the identification of anxiety symptoms or depressive symptoms using a self-report questionnaire is likely to be subjective so response bias is possible, although the questionnaires used had good validity and reliability. Further objective measures might be needed to confirm the diagnoses. Lastly, longitudinal follow-up studies are required to track the evolution of symptoms and measure the long-term mental health impacts of the COVID-19 pandemic among medical students worldwide.

## Conclusions

This systematic review and meta-analysis highlighted that medical students are at high risk of developing anxiety and depression during the COVID-19 pandemic. A substantial proportion of medical students have experienced adverse pandemic-related psychological impacts. Appropriate strategies are needed to meet the psychological needs of this population and protect their mental health status. Future studies are necessary to assess the appropriateness of management strategies to treat and prevent mental health disorders among medical students during the COVID-19 pandemic.

### Supplementary Information

Additional file 1: Appendix 1. Search Strategy Protocol.

Additional file 2: Appendix 2. PRISMA 2020 Checklist.

Additional file 3: JBI critical appraisal checklist for studies reporting prevalence data.

## Data Availability

Not applicable.

## Abbreviation

COVID-19: Coronavirus disease 2019
PRISMA: Preferred Reporting Items for Systematic Reviews and Meta-Analyses
M.D: Doctor of Medicine
M.B.B.S: Bachelor of Medicine and Bachelor of Surgery
REML: Restricted maximum likelihood
MS: Moderate and severe
GAD-7: Generalized Anxiety Disorder-7
BAI: Beck Anxiety Inventory
STAI-6: State-Trait Anxiety Inventory-6
PHQ-9: Patients Health Questionnaire-9
SAS: Zung Self-rating Anxiety Scale
SDS: Zung Self-rating Depression Scale
BDI-II: Beck Depression Inventory-II
HADS: Hospital Anxiety and Depression Scale
DASS: Depression, Anxiety and Stress Scale
CES-D: Center for Epidemiology Studies for Depression scale
VAS: Visual Analogue Scale
