# Supplementary material for: Global prevalence of anxiety and depression among medical students during the COVID-19 pandemic: a systematic review and meta-analysis
Source: BMC Psychol. 2024 Jun 10;12:338. doi: 10.1186/s40359-024-01838-y (PMC11163725; doi:10.1186/s40359-024-01838-y)
Supplement: Supplementary file 1 — Additional file 1: Appendix 1. Search Strategy Protocol. [file 40359_2024_1838_MOESM1_ESM.docx]

**Appendix 1 Search Strategy Protocol**

Search strategy of mental health outcome among medical and nursing students during COVID-19 pandemic

| **Source** | **Search strategy** | **Hits retrieved** |
| --- | --- | --- |
| CINAHL | Medical students;ab OR medicine students;ab OR students in medicine;ab  AND anxiety;ab OR anxiety disorder;ab OR generalized anxiety disorder;ab OR depression;ab OR depressive disorder;ab OR depressive symptom;ab  AND COVID-19;ab OR coronavirus disease 2019;ab OR pandemic;ab OR 2019-ncov;ab OR sars-cov-2;ab OR cov-19;ab | 225 |
| EMBASE | ('depression'/exp OR depression) OR ('anxiety'/exp OR anxiety) OR ('mental disorder*':ab,ti OR 'mental health*':ab,ti OR anxiety:ab,ti OR 'anxiety disorder*':ab,ti OR anxiety*:ab,ti OR depress*:ab,ti OR depressed:ab,ti OR depression:ab,ti OR 'depressive disorder':ab,ti OR 'depressive disorder, major':ab,ti OR 'major depression':ab,ti OR 'major depressive disorder':ab,ti OR mdd:ab,ti OR sadness:ab,ti) AND ('clinical student*':ab,ti OR 'medical student*':ab,ti OR 'med student*':ab,ti OR 'medical trainee*':ab,ti OR 'preclinical student*':ab,ti OR 'student doctor*':ab,ti OR 'student physician*':ab,ti OR 'undergraduate medic*':ab,ti OR 'education, medical, undergraduate':ab,ti OR 'education, medical':ab,ti OR 'medical postgraduate*':ab,ti OR 'medical graduate*':ab,ti) AND ('covid 19':ab,ti OR 'coronavirus disease 2019':ab,ti OR pandemic:ab,ti OR '2019 ncov':ab,ti OR 'sars cov 2':ab,ti OR 'cov 19':ab,ti) | 581 |
| MEDLINE complete | AB mental disorder OR AB mental health OR AB affective disorder OR AB mood disorder OR AB depressive disorder OR AB depression OR AB anxiety OR AB stress, psychological OR AB depress* OR AB anxiety* OR AB mental wellbeing  AND AB medical student* OR AB medical undergraduate* OR AB medical postgraduate OR AB education, medical, undergraduate OR AB education, medical  AND AB COVID-19 OR AB coronavirus disease 2019 OR AB pandemic OR AB 2019-ncov OR AB sars-cov-2 OR AB cov-19 | 1010 |
| PubMed | (((("mental disorder"[Title/Abstract] OR "mental health"[Title/Abstract] OR "affective disorder"[Title/Abstract] OR "mood disorder"[Title/Abstract] OR "depressive disorder"[MeSH Terms] OR "depression"[MeSH Terms] OR "anxiety"[MeSH Terms] OR "stress, psychological"[MeSH Terms] OR "depress*"[Title/Abstract] OR "anxiety*"[Title/Abstract] OR "mental wellbeing"[Title/Abstract])) AND (("medical student*"[Title/Abstract] OR "medical undergraduate*"[Title/Abstract] OR "medical postgraduate"[Title/Abstract] OR "education, medical, undergraduate"[MeSH Terms] OR "education, medical"[MeSH Terms]))) AND ((((((COVID-19[Title/Abstract]) OR (coronavirus disease 2019[Title/Abstract])) OR (pandemic[Title/Abstract])) OR (2019-ncov[Title/Abstract])) OR (sars-cov-2[Title/Abstract])) OR (cov-19[Title/Abstract])) | 642 |
| Web Of Science | mood disorder (TS) or mood disorder* (TS) or affective disorder* (TS) or mental disorder* (TS) or mental health* (TS) or anxiety* (TS) or Depress* (TS) or Depression (TS) or mental wellbeing (TS)  AND medical student* (TS) or medical postgraduate* (TS) or medical graduate* (TS) or medical undergraduate* (TS)  AND COVID-19 (TS) or coronavirus disease 2019 (TS) or pandemic (TS) or 2019 ncov (TS) or sars cov 2 (TS) or cov 19 (TS) | 686 |
