# Supplementary material for: Global prevalence of anxiety and depression among medical students during the COVID-19 pandemic: a systematic review and meta-analysis
Source: BMC Psychol. 2024 Jun 10;12:338. doi: 10.1186/s40359-024-01838-y (PMC11163725; doi:10.1186/s40359-024-01838-y)
Supplement: Supplementary file 3 — Additional file 3: JBI critical appraisal checklist for studies reporting prevalence data. [file 40359_2024_1838_MOESM3_ESM.docx]

| **Appendix 3 JBI critical appraisal checklist for studies reporting prevalence data** | | | | | | | | | | | |  |  |  |  |
| --- | --- | --- | --- | --- | --- | --- | --- | --- | --- | --- | --- | --- | --- | --- | --- |
| **No** | **Author/year** | **Was the sample frame appropriate to address the target population?** | **Were study participants sampled in an appropriate way?** | **Was the sample size adequate?** | **Were the study subjects and the setting described in detail?** | | **Was the data analysis conducted with sufficient coverage of the identified sample?** | **Were valid methods used for the identification of the condition?** | **Was the condition measured in a standard, reliable way for all participants?** | **Was there appropriate statistical analysis?** | **Was the response rate adequate, and if not, was the low response rate managed appropriately?** | |  |  |  |
|  |  |  |  |  |  |  |  |  |  |  |  |  |  |  |  |
|  |  |  |  |  |  |  |  |  |  |  |  |  |  |  |  |
| 1 | AbuDujain et al., 2021 | Y | Y | Y | Y | | Y | Y | Y | Y | Y | |  |  |  |
|  |  |  |  |  |  |  |  |  |  |  |  |  |  |  |  |
| 2 | Adhikari et al., 2021 | Y | Y | Y | Y | | Y | Y | Y | Y | Y | |  |  |  |
|  |  |  |  |  |  |  |  |  |  |  |  |  |  |  |  |
| 3 | Aftab et al., 2021 | Y | Y | Y | Y | | Y | Y | Y | Y | Y | |  |  |  |
|  |  |  |  |  |  |  |  |  |  |  |  |  |  |  |  |
| 4 | Ahmed et al., 2020 | Y | Y | Y | Y | | Y | Y | Y | Y | Y | |  |  |  |
|  |  |  |  |  |  |  |  |  |  |  |  |  |  |  |  |
| 5 | Al-Hasani et al., 2021 | Y | Y | Y | Y | | Y | Y | Y | Y | Y | |  |  |  |
|  |  |  |  |  |  |  |  |  |  |  |  |  |  |  |  |
| 6 | Ali et al., 2022 | Y | Y | Y | Y | | Y | Y | Y | Y | Y | |  |  |  |
|  |  |  |  |  |  |  |  |  |  |  |  |  |  |  |  |
| 7 | Alkhamees et al., 2020 | Y | Y | Y | Y | | Y | Y | Y | Y | Y | |  |  |  |
|  |  |  |  |  |  |  |  |  |  |  |  |  |  |  |  |
| 8 | Alkwai, 2021 | Y | Y | Y | U | | Y | Y | Y | Y | Y | |  |  |  |
|  |  |  |  |  |  |  |  |  |  |  |  |  |  |  |  |
| 9 | Allah et al., 2021 | Y | Y | Y | Y | | Y | Y | Y | Y | Y | |  |  |  |
|  |  |  |  |  |  |  |  |  |  |  |  |  |  |  |  |
| 10 | Almarri et al., 2022 | Y | Y | Y | Y | | Y | Y | Y | Y | Y | |  |  |  |
|  |  |  |  |  |  |  |  |  |  |  |  |  |  |  |  |
| 11 | Almutairi A., Jahan S., 2022 | Y | Y | Y | Y | | Y | Y | Y | Y | Y | |  |  |  |
|  |  |  |  |  |  |  |  |  |  |  |  |  |  |  |  |
| 12 | Alrashed et al., 2022 | Y | Y | Y | Y | | Y | Y | Y | Y | Y | |  |  |  |
|  |  |  |  |  |  |  |  |  |  |  |  |  |  |  |  |
| 13 | Alrashed et al., 2021 | Y | Y | Y | Y | | Y | Y | Y | Y | Y | |  |  |  |
|  |  |  |  |  |  |  |  |  |  |  |  |  |  |  |  |
| 14 | Aolymat et al., 2023 | Y | Y | Y | Y | | Y | Y | Y | Y | Y | |  |  |  |
|  |  |  |  |  |  |  |  |  |  |  |  |  |  |  |  |
| 15 | Avila-Carrasco et al., 2022 | Y | Y | Y | U | | Y | Y | Y | Y | Y | |  |  |  |
|  |  |  |  |  |  |  |  |  |  |  |  |  |  |  |  |
| 16 | Batais et al., 2021 | Y | Y | Y | Y | | Y | Y | Y | Y | Y | |  |  |  |
|  |  |  |  |  |  |  |  |  |  |  |  |  |  |  |  |
| 17 | Bilgi et al., 2021 | Y | Y | Y | N | | Y | Y | Y | Y | Y | |  |  |  |
|  |  |  |  |  |  |  |  |  |  |  |  |  |  |  |  |
| 18 | Biswas et al., 2022 | Y | Y | Y | Y | | Y | Y | Y | Y | Y | |  |  |  |
|  |  |  |  |  |  |  |  |  |  |  |  |  |  |  |  |
| 19 | Bolatov et al., 2021 | Y | Y | Y | Y | | Y | Y | Y | Y | Y | |  |  |  |
|  |  |  |  |  |  |  |  |  |  |  |  |  |  |  |  |
| 20 | Cao et al., 2020 | Y | Y | Y | Y | | Y | Y | Y | Y | Y | |  |  |  |
|  |  |  |  |  |  |  |  |  |  |  |  |  |  |  |  |
| 21 | Capdevila-Gaudens et al., 2021 | Y | Y | Y | N | | Y | Y | Y | Y | Y | |  |  |  |
|  |  |  |  |  |  |  |  |  |  |  |  |  |  |  |  |
| 22 | Carletto et al., 2022 | Y | Y | Y | Y | | Y | Y | Y | Y | Y | |  |  |  |
|  |  |  |  |  |  |  |  |  |  |  |  |  |  |  |  |
| 23 | Chakeeyanun et al., 2023 | Y | Y | Y | Y | | Y | Y | Y | Y | Y | |  |  |  |
|  |  |  |  |  |  |  |  |  |  |  |  |  |  |  |  |
| 24 | Chang et al., 2021 | Y | Y | Y | Y | | Y | Y | Y | Y | Y | |  |  |  |
|  |  |  |  |  |  |  |  |  |  |  |  |  |  |  |  |
| 25 | Chaudhuri et al., 2020 | Y | Y | Y | Y | | Y | Y | Y | Y | Y | |  |  |  |
|  |  |  |  |  |  |  |  |  |  |  |  |  |  |  |  |
| 26 | Chootong et al., 2022 | Y | Y | Y | Y | | Y | Y | Y | Y | Y | |  |  |  |
|  |  |  |  |  |  |  |  |  |  |  |  |  |  |  |  |
| 27 | Christophers et al., 2021 | Y | Y | Y | Y | | Y | Y | Y | Y | Y | |  |  |  |
|  |  |  |  |  |  |  |  |  |  |  |  |  |  |  |  |
| 28 | Çimen İ et al., 2022 | Y | Y | Y | U | | Y | Y | Y | Y | Y | |  |  |  |
|  |  |  |  |  |  |  |  |  |  |  |  |  |  |  |  |
| 29 | Cinar Tanriverdi et al., 2023 | Y | Y | Y | Y | | Y | Y | Y | Y | Y | |  |  |  |
|  |  |  |  |  |  |  |  |  |  |  |  |  |  |  |  |
| 30 | de Souza et al., 2021 | Y | Y | Y | Y | | Y | Y | Y | Y | Y | |  |  |  |
|  |  |  |  |  |  |  |  |  |  |  |  |  |  |  |  |
| 31 | Deb N., Roy P., 2022 | Y | Y | Y | Y | | Y | Y | Y | Y | Y | |  |  |  |
|  |  |  |  |  |  |  |  |  |  |  |  |  |  |  |  |
| 32 | Deng et al., 2021 | Y | Y | Y | Y | | Y | Y | Y | Y | Y | |  |  |  |
|  |  |  |  |  |  |  |  |  |  |  |  |  |  |  |  |
| 33 | Ecker et al., 2022 | Y | Y | Y | Y | | Y | Y | Y | Y | Y | |  |  |  |
|  |  |  |  |  |  |  |  |  |  |  |  |  |  |  |  |
| 34 | Eid et al., 2021 | Y | Y | Y | Y | | Y | Y | Y | Y | Y | |  |  |  |
|  |  |  |  |  |  |  |  |  |  |  |  |  |  |  |  |
| 35 | Eleftheriou et al., 2021 | Y | Y | Y | Y | | Y | Y | Y | Y | Y | |  |  |  |
|  |  |  |  |  |  |  |  |  |  |  |  |  |  |  |  |
| 36-1 | Ertek et al., 2022 | Y | Y | Y | Y | | Y | Y | Y | Y | Y | |  |  |  |
|  |  |  |  |  |  |  |  |  |  |  |  |  |  |  |  |
| 36-2 | Ertek et al., 2022 | Y | Y | Y | Y | | Y | Y | Y | Y | Y | |  |  |  |
|  |  |  |  |  |  |  |  |  |  |  |  |  |  |  |  |
| 37 | Esmat et al., 2021 | Y | Y | Y | Y | | Y | Y | Y | Y | Y | |  |  |  |
|  |  |  |  |  |  |  |  |  |  |  |  |  |  |  |  |
| 38 | Essadek et al., 2022 | Y | Y | Y | U | | Y | Y | Y | Y | Y | |  |  |  |
|  |  |  |  |  |  |  |  |  |  |  |  |  |  |  |  |
| 39 | Essangri et al., 2021 | Y | Y | Y | Y | | Y | Y | Y | Y | Y | |  |  |  |
|  |  |  |  |  |  |  |  |  |  |  |  |  |  |  |  |
| 40 | Frajerman et al., 2022 | Y | Y | Y | Y | | Y | Y | Y | Y | Y | |  |  |  |
|  |  |  |  |  |  |  |  |  |  |  |  |  |  |  |  |
| Gao et al., 2021 | Y | Y | Y | Y | |  | Y | Y | Y | Y |  |  | Y | |  |
|  |  |  |  |  |  |  |  |  |  |  |  |  |  |  |  |
| 41-2 | Gao et al., 2021 | Y | Y | Y | Y | | Y | Y | Y | Y | Y | |  |  |  |
|  |  |  |  |  |  |  |  |  |  |  |  |  |  |  |  |
| 42 | Gómez-Durán et al., 2022 | Y | Y | Y | Y | | Y | Y | Y | Y | Y | |  |  |  |
|  |  |  |  |  |  |  |  |  |  |  |  |  |  |  |  |
| 43 | Guo et al., 2021 | Y | Y | Y | Y | | Y | Y | Y | Y | Y | |  |  |  |
|  |  |  |  |  |  |  |  |  |  |  |  |  |  |  |  |
| 44 | Guo et al., 2021 | Y | Y | Y | Y | | Y | Y | Y | Y | Y | |  |  |  |
|  |  |  |  |  |  |  |  |  |  |  |  |  |  |  |  |
| 45 | Gupta et al., 2022 | Y | Y | U | Y | | Y | Y | Y | Y | Y | |  |  |  |
|  |  |  |  |  |  |  |  |  |  |  |  |  |  |  |  |
| 46 | Guse et al., 2021 | Y | Y | Y | Y | | Y | Y | Y | Y | Y | |  |  |  |
|  |  |  |  |  |  |  |  |  |  |  |  |  |  |  |  |
| 47 | Halperin et al., 2021 | Y | Y | U | Y | | Y | Y | Y | Y | Y | |  |  |  |
|  |  |  |  |  |  |  |  |  |  |  |  |  |  |  |  |
| 48 | Hassnain et al., 2021 | Y | Y | N | Y | | Y | Y | Y | Y | Y | |  |  |  |
|  |  |  |  |  |  |  |  |  |  |  |  |  |  |  |  |
| 49 | Hjiej et al., 2022 | Y | Y | Y | Y | | Y | Y | Y | Y | Y | |  |  |  |
|  |  |  |  |  |  |  |  |  |  |  |  |  |  |  |  |
| 50 | Huarcaya-Victoria et al., 2021 | Y | Y | Y | Y | | Y | Y | Y | Y | Y | |  |  |  |
|  |  |  |  |  |  |  |  |  |  |  |  |  |  |  |  |
| 51 | Ismail et al., 2021 | Y | Y | U | Y | | Y | Y | Y | Y | Y | |  |  |  |
|  |  |  |  |  |  |  |  |  |  |  |  |  |  |  |  |
| 52 | Jindal et al., 2020 | Y | Y | Y | Y | | Y | Y | Y | Y | Y | |  |  |  |
|  |  |  |  |  |  |  |  |  |  |  |  |  |  |  |  |
| 53 | Junaid Tahir et al., 2022 | Y | Y | U | Y | | Y | Y | Y | Y | Y | |  |  |  |
|  |  |  |  |  |  |  |  |  |  |  |  |  |  |  |  |
| 54 | Jupina et al., 2022 | Y | Y | Y | Y | | Y | Y | Y | Y | Y | |  |  |  |
|  |  |  |  |  |  |  |  |  |  |  |  |  |  |  |  |
| 55 | Kamran et al., 2022 | Y | Y | Y | Y | | Y | Y | Y | Y | Y | |  |  |  |
|  |  |  |  |  |  |  |  |  |  |  |  |  |  |  |  |
| 56 | Khidri et al., 2022 | Y | Y | Y | Y | | Y | Y | Y | Y | Y | |  |  |  |
|  |  |  |  |  |  |  |  |  |  |  |  |  |  |  |  |
| 57 | Kim et al., 2022 | Y | Y | Y | Y | | Y | Y | Y | Y | Y | |  |  |  |
|  |  |  |  |  |  |  |  |  |  |  |  |  |  |  |  |
| 58 | Kuman Tunçel et al., 2021 | Y | Y | Y | Y | | Y | Y | Y | Y | Y | |  |  |  |
|  |  |  |  |  |  |  |  |  |  |  |  |  |  |  |  |
| 59 | Kumar et al., 2021 | Y | Y | U | Y | | Y | Y | Y | Y | Y | |  |  |  |
|  |  |  |  |  |  |  |  |  |  |  |  |  |  |  |  |
| 60 | Lee et al., 2021 | Y | Y | U | Y | | Y | Y | Y | Y | Y | |  |  |  |
|  |  |  |  |  |  |  |  |  |  |  |  |  |  |  |  |
| 61 | Leroy et al., 2021 | Y | Y | Y | Y | | Y | Y | Y | Y | Y | |  |  |  |
|  |  |  |  |  |  |  |  |  |  |  |  |  |  |  |  |
| 62 | Liu et al., 2021 | Y | Y | Y | Y | | Y | Y | Y | Y | Y | |  |  |  |
|  |  |  |  |  |  |  |  |  |  |  |  |  |  |  |  |
| 63 | Liu et al., 2020 | Y | Y | Y | Y | | Y | Y | Y | Y | Y | |  |  |  |
|  |  |  |  |  |  |  |  |  |  |  |  |  |  |  |  |
| 64 | Lu et al., 2022 | Y | Y | Y | Y | | Y | Y | Y | Y | Y | |  |  |  |
|  |  |  |  |  |  |  |  |  |  |  |  |  |  |  |  |
| 65 | Madaan et al., 2022 | Y | Y | Y | Y | | Y | Y | Y | Y | Y | |  |  |  |
|  |  |  |  |  |  |  |  |  |  |  |  |  |  |  |  |
| 66 | Manjareeka M., Pathak M., 2021 | Y | Y | Y | Y | | Y | Y | Y | Y | Y | |  |  |  |
|  |  |  |  |  |  |  |  |  |  |  |  |  |  |  |  |
| 67 | Maroof et al., 2022 | Y | Y | Y | Y | | Y | Y | Y | Y | Y | |  |  |  |
|  |  |  |  |  |  |  |  |  |  |  |  |  |  |  |  |
| 68 | Mendes et al., 2021 | Y | Y | U | Y | | Y | Y | Y | Y | Y | |  |  |  |
|  |  |  |  |  |  |  |  |  |  |  |  |  |  |  |  |
| 69 | Meng et al., 2021 | Y | Y | N | Y | | Y | Y | Y | Y | Y | |  |  |  |
|  |  |  |  |  |  |  |  |  |  |  |  |  |  |  |  |
| 70 | Mishra et al., 2023 | Y | Y | Y | Y | | Y | Y | Y | Y | Y | |  |  |  |
|  |  |  |  |  |  |  |  |  |  |  |  |  |  |  |  |
| 71 | Mishra et al., 2022 | Y | Y | Y | Y | | Y | Y | Y | Y | Y | |  |  |  |
|  |  |  |  |  |  |  |  |  |  |  |  |  |  |  |  |
| 72 | Mohamed et al., 2022 | Y | Y | N | Y | | Y | Y | Y | Y | Y | |  |  |  |
|  |  |  |  |  |  |  |  |  |  |  |  |  |  |  |  |
| 73 | Muhammad Alfareed Zafar et al., 2020 | Y | Y | Y | Y | | Y | Y | Y | Y | Y | |  |  |  |
|  |  |  |  |  |  |  |  |  |  |  |  |  |  |  |  |
| 74 | Nakhostin-Ansari et al., 2020 | Y | Y | Y | Y | | Y | Y | Y | Y | Y | |  |  |  |
|  |  |  |  |  |  |  |  |  |  |  |  |  |  |  |  |
| 75 | Natalia D., Syakurah R.A., 2021 | Y | Y | Y | Y | | Y | Y | Y | Y | Y | |  |  |  |
|  |  |  |  |  |  |  |  |  |  |  |  |  |  |  |  |
| 76 | Nguyen et al., 2022 | Y | Y | Y | Y | | Y | Y | Y | Y | Y | |  |  |  |
|  |  |  |  |  |  |  |  |  |  |  |  |  |  |  |  |
| 77 | Ni et al., 2021 | Y | Y | Y | Y | | Y | Y | Y | Y | Y | |  |  |  |
|  |  |  |  |  |  |  |  |  |  |  |  |  |  |  |  |
| 78 | Nihmath Nisha et al., 2020 | Y | Y | Y | Y | | Y | Y | Y | Y | Y | |  |  |  |
|  |  |  |  |  |  |  |  |  |  |  |  |  |  |  |  |
| 79 | Nishimura et al., 2021 | Y | Y | U | Y | | Y | Y | Y | Y | Y | |  |  |  |
|  |  |  |  |  |  |  |  |  |  |  |  |  |  |  |  |
| 80 | Nugraha et al., 2023 | Y | Y | Y | Y | | Y | Y | Y | Y | Y | |  |  |  |
|  |  |  |  |  |  |  |  |  |  |  |  |  |  |  |  |
| 81 | Pattanaseri et al., 2022 | Y | Y | Y | Y | | Y | Y | Y | Y | Y | |  |  |  |
|  |  |  |  |  |  |  |  |  |  |  |  |  |  |  |  |
| 82 | Paz et al., 2023 | Y | Y | Y | Y | | Y | Y | Y | Y | Y | |  |  |  |
|  |  |  |  |  |  |  |  |  |  |  |  |  |  |  |  |
| 83 | Pedraz-Petrozzi et al., 2021 | Y | Y | Y | Y | | Y | Y | Y | Y | Y | |  |  |  |
|  |  |  |  |  |  |  |  |  |  |  |  |  |  |  |  |
| 84 | Pelaccia et al., 2021 | Y | Y | Y | Y | | Y | Y | Y | Y | Y | |  |  |  |
|  |  |  |  |  |  |  |  |  |  |  |  |  |  |  |  |
| 85 | Peng et al., 2022 | Y | Y | Y | Y | | Y | Y | Y | Y | Y | |  |  |  |
|  |  |  |  |  |  |  |  |  |  |  |  |  |  |  |  |
| 86 | Perissotto et al., 2021 | Y | Y | Y | Y | | Y | Y | Y | Y | Y | |  |  |  |
|  |  |  |  |  |  |  |  |  |  |  |  |  |  |  |  |
| 87 | Poon et al., 2021 | Y | Y | Y | Y | | Y | Y | Y | Y | Y | |  |  |  |
|  |  |  |  |  |  |  |  |  |  |  |  |  |  |  |  |
| 88 | Ravikumar et al., 2022 | Y | Y | Y | Y | | Y | Y | Y | Y | Y | |  |  |  |
|  |  |  |  |  |  |  |  |  |  |  |  |  |  |  |  |
| 89 | Reddy C.R.E.T., Tekulapally K., 2022 | Y | Y | U | Y | | Y | Y | Y | Y | Y | |  |  |  |
|  |  |  |  |  |  |  |  |  |  |  |  |  |  |  |  |
| 90 | Rehman et al., 2022 | Y | Y | N | Y | | Y | Y | Y | Y | Y | |  |  |  |
|  |  |  |  |  |  |  |  |  |  |  |  |  |  |  |  |
| 91 | Risal et al., 2020 | Y | Y | U | Y | | Y | Y | Y | Y | Y | |  |  |  |
|  |  |  |  |  |  |  |  |  |  |  |  |  |  |  |  |
| 92 | Rolland et al., 2022 | Y | Y | Y | Y | | Y | Y | Y | Y | Y | |  |  |  |
|  |  |  |  |  |  |  |  |  |  |  |  |  |  |  |  |
| 93 | Rutkowska et al., 2021 | Y | Y | Y | Y | | Y | Y | Y | Y | Y | |  |  |  |
|  |  |  |  |  |  |  |  |  |  |  |  |  |  |  |  |
| 94 | Saali et al., 2022 | Y | Y | Y | Y | | Y | Y | Y | Y | Y | |  |  |  |
|  |  |  |  |  |  |  |  |  |  |  |  |  |  |  |  |
| 95 | Saddik et al., 2020 | Y | Y | Y | Y | | Y | Y | Y | Y | Y | |  |  |  |
|  |  |  |  |  |  |  |  |  |  |  |  |  |  |  |  |
| 96 | Saeed N., Javed N., 2021 | Y | Y | Y | Y | | Y | Y | Y | Y | Y | |  |  |  |
|  |  |  |  |  |  |  |  |  |  |  |  |  |  |  |  |
| 97 | Safa et al., 2021 | Y | Y | Y | Y | | Y | Y | Y | Y | Y | |  |  |  |
|  |  |  |  |  |  |  |  |  |  |  |  |  |  |  |  |
| 98 | Saguem et al., 2022 | Y | Y | Y | Y | | Y | Y | Y | Y | Y | |  |  |  |
|  |  |  |  |  |  |  |  |  |  |  |  |  |  |  |  |
| 99 | Santander-Hernández et al., 2022 | Y | Y | Y | Y | | Y | Y | Y | Y | Y | |  |  |  |
|  |  |  |  |  |  |  |  |  |  |  |  |  |  |  |  |
| 100 | Saravia-Bartra et al., 2020 | Y | Y | Y | Y | | Y | Y | Y | Y | Y | |  |  |  |
|  |  |  |  |  |  |  |  |  |  |  |  |  |  |  |  |
|  |  |  |  |  |  |  |  |  |  |  |  |  |  |  |  |
| 101 | Sartorão Filho et al., 2020 | Y | Y | Y | Y | | Y | Y | Y | Y | Y | |  |  |  |
|  |  |  |  |  |  |  |  |  |  |  |  |  |  |  |  |
| 102 | Selvamani et al., 2022 | Y | Y | Y | Y | | Y | Y | Y | Y | Y | |  |  |  |
|  |  |  |  |  |  |  |  |  |  |  |  |  |  |  |  |
| 103 | Shailaja et al., 2020 | Y | Y | Y | Y | | Y | Y | Y | Y | Y | |  |  |  |
|  |  |  |  |  |  |  |  |  |  |  |  |  |  |  |  |
| 104 | Shreevastava et al., 2022 | Y | Y | Y | Y | | Y | Y | Y | Y | Y | |  |  |  |
|  |  |  |  |  |  |  |  |  |  |  |  |  |  |  |  |
| 105 | Soltan et al., 2021 | Y | Y | Y | Y | | Y | Y | Y | Y | Y | |  |  |  |
|  |  |  |  |  |  |  |  |  |  |  |  |  |  |  |  |
| 106 | Song et al., 2022 | Y | Y | Y | Y | | Y | Y | Y | Y | Y | |  |  |  |
|  |  |  |  |  |  |  |  |  |  |  |  |  |  |  |  |
| 107 | Srivastava et al., 2021 | Y | Y | Y | Y | | Y | Y | Y | Y | Y | |  |  |  |
|  |  |  |  |  |  |  |  |  |  |  |  |  |  |  |  |
| 108-1 | Stanislawski et al., 2023 | Y | Y | Y | Y | | Y | Y | Y | Y | Y | |  |  |  |
|  |  |  |  |  |  |  |  |  |  |  |  |  |  |  |  |
| 108-2 | Stanislawski et al., 2023 | Y | Y | Y | Y | | Y | Y | Y | Y | Y | |  |  |  |
|  |  |  |  |  |  |  |  |  |  |  |  |  |  |  |  |
| 108-3 | Stanislawski et al., 2023 | Y | Y | Y | Y | | Y | Y | Y | Y | Y | |  |  |  |
|  |  |  |  |  |  |  |  |  |  |  |  |  |  |  |  |
| 108-4 | Stanislawski et al., 2023 | Y | Y | Y | Y | | Y | Y | Y | Y | Y | |  |  |  |
|  |  |  |  |  |  |  |  |  |  |  |  |  |  |  |  |
| 109 | Sudi et al., 2022 | Y | Y | Y | Y | | Y | Y | Y | Y | Y | |  |  |  |
|  |  |  |  |  |  |  |  |  |  |  |  |  |  |  |  |
| 110 | Tee et al., 2022 | Y | Y | Y | Y | | Y | Y | Y | Y | Y | |  |  |  |
|  |  |  |  |  |  |  |  |  |  |  |  |  |  |  |  |
| 111 | Teh et al., 2023 | Y | Y | Y | Y | | Y | Y | Y | Y | Y | |  |  |  |
|  |  |  |  |  |  |  |  |  |  |  |  |  |  |  |  |
| 112 | Tejoyuwono et al., 2021 | Y | Y | Y | Y | | Y | Y | Y | Y | Y | |  |  |  |
|  |  |  |  |  |  |  |  |  |  |  |  |  |  |  |  |
| 113 | Vala et al., 2020 | Y | Y | Y | Y | | Y | Y | Y | Y | Y | |  |  |  |
|  |  |  |  |  |  |  |  |  |  |  |  |  |  |  |  |
| 114 | Wu et al., 2022 | Y | Y | Y | Y | | Y | Y | Y | Y | Y | |  |  |  |
|  |  |  |  |  |  |  |  |  |  |  |  |  |  |  |  |
| 115 | Xiang et al, 2022 | Y | Y | Y | Y | | Y | Y | Y | Y | Y | |  |  |  |
|  |  |  |  |  |  |  |  |  |  |  |  |  |  |  |  |
| 116 | Xiao et al., 2020 | Y | Y | Y | Y | | Y | Y | Y | Y | Y | |  |  |  |
|  |  |  |  |  |  |  |  |  |  |  |  |  |  |  |  |
| 117 | Xie et al., 2021 | Y | Y | Y | Y | | Y | Y | Y | Y | Y | |  |  |  |
|  |  |  |  |  |  |  |  |  |  |  |  |  |  |  |  |
| 118 | Xiong et al., 2021 | Y | Y | Y | Y | | Y | Y | Y | Y | Y | |  |  |  |
|  |  |  |  |  |  |  |  |  |  |  |  |  |  |  |  |
| 119 | Yang et al., 2022 | Y | Y | Y | Y | | Y | Y | Y | Y | Y | |  |  |  |
|  |  |  |  |  |  |  |  |  |  |  |  |  |  |  |  |
| 120 | Yang et al., 2022 | Y | Y | Y | Y | | Y | Y | Y | Y | Y | |  |  |  |
|  |  |  |  |  |  |  |  |  |  |  |  |  |  |  |  |
| 121 | Yin et al., 2021 | Y | Y | Y | Y | | Y | Y | Y | Y | Y | |  |  |  |
|  |  |  |  |  |  |  |  |  |  |  |  |  |  |  |  |
| 122 | Yuan et al., 2021 | Y | Y | Y | Y | | Y | Y | Y | Y | Y | |  |  |  |
|  |  |  |  |  |  |  |  |  |  |  |  |  |  |  |  |
| 123 | Yun et al., 2021 | Y | Y | Y | U | | Y | Y | Y | Y | Y | |  |  |  |
|  |  |  |  |  |  |  |  |  |  |  |  |  |  |  |  |
| 124 | Zhang et al., 2021 | Y | Y | Y | Y | | Y | Y | Y | Y | Y | |  |  |  |
|  |  |  |  |  |  |  |  |  |  |  |  |  |  |  |  |
| 125 | Zhang et al., 2021 | Y | Y | Y | Y | | Y | Y | Y | Y | Y | |  |  |  |
|  |  |  |  |  |  |  |  |  |  |  |  |  |  |  |  |
| 126 | Zhang et al., 2021 | Y | Y | Y | Y | | Y | Y | Y | Y | Y | |  |  |  |
|  |  |  |  |  |  |  |  |  |  |  |  |  |  |  |  |
| 127 | Zhao et al., 2022 | Y | Y | Y | Y | | Y | Y | Y | Y | Y | |  |  |  |
|  |  |  |  |  |  |  |  |  |  |  |  |  |  |  |  |
| 128 | Zhao et al., 2021 | Y | Y | Y | Y | | Y | Y | Y | Y | Y | |  |  |  |
|  |  |  |  |  |  |  |  |  |  |  |  |  |  |  |  |
| 129 | Zheng et al., 2021 | Y | Y | Y | Y | | Y | Y | Y | Y | Y | |  |  |  |
|  |  |  |  |  |  |  |  |  |  |  |  |  |  |  |  |
| 130 | Zhong et al., 2021 | Y | Y | Y | Y | | Y | Y | Y | Y | Y | |  |  |  |
|  |  |  |  |  |  |  |  |  |  |  |  |  |  |  |  |
| N, No; Y, Yes; U, Unclear | | | | | |  |  |  |  |  |  |  |  |  |  |
